# Insights into bioactive constituents of onion (*Allium cepa* L.) waste: a comparative metabolomics study enhanced by chemometric tools

**DOI:** 10.1186/s12906-024-04559-2

**Published:** 2024-07-15

**Authors:** Mariam M. Elattar, Hala M. Hammoda, Doaa A. Ghareeb, Shaymaa A. Abdulmalek, Fatma. A. Abdelrahim, Inas A. K. Seif, Hend M. Dawood, Reham S. Darwish

**Affiliations:** 1 Department of Pharmacognosy, Faculty of Pharmacy, Alexandria University, Alexandria, 21521 Egypt; 2 Bio-screening and Preclinical Trial Lab, Biochemistry Department, Faculty of Science, Alexandria University, Alexandria, Egypt; 3 Center of Excellence for Drug Preclinical Studies (CE-DPS), Pharmaceutical and Fermentation Industry Development Center, City of Scientific Research & Technological Applications (SRTA- city), New Borg El Arab, Alexandria, Egypt; 4 Research Projects Unit, Pharos University, Alexandria, Egypt

**Keywords:** Onion, High-value waste, Chemical profiling, PDE-5 inhibitory activity, Anti-inflammatory activity, Biomarkers

## Abstract

**Background:**

Onion waste was reported to be a valuable source of bioactive constituents with potential health-promoting benefits. This sparked a surge of interest among scientists for its valorization. This study aims to investigate the chemical profiles of peel and root extracts of four onion cultivars (red, copper-yellow, golden yellow and white onions) and evaluate their erectogenic and anti-inflammatory potentials.

**Methods:**

UPLC-QqQ-MS/MS analysis and chemometric tools were utilized to determine the chemical profiles of onion peel and root extracts. The erectogenic potential of the extracts was evaluated using the PDE-5 inhibitory assay, while their anti-inflammatory activity was determined by identifying their downregulating effect on the gene expression of IL-6, IL-1*β*, IFN-*γ*, and TNF-*α* in LPS-stimulated WBCs.

**Results:**

A total of 103 metabolites of diverse chemical classes were identified, with the most abundant being flavonoids. The organ’s influence on the chemical profiles of the samples outweighed the influence of the cultivar, as evidenced by the close clustering of samples from the same organ compared to the distinct separation of root and peel samples from the same cultivar. Furthermore, the tested extracts demonstrated promising PDE-5 and anti-inflammatory potentials and effectively suppressed the upregulation of pro-inflammatory markers in LPS-stimulated WBCs. The anti-inflammatory activities exerted by peel samples surpassed those of root samples, highlighting the importance of selecting the appropriate organ to maximize activity. The main metabolites correlated with PDE-5 inhibition were cyanidin 3-*O*-(malonyl-acetyl)-glucoside and quercetin dimer hexoside, while those correlated with IL-1*β* inhibition were *γ*-glutamyl-methionine sulfoxide, *γ*-glutamyl glutamine, sativanone, and stearic acid. Taxifolin, 3’-hydroxymelanettin, and oleic acid were highly correlated with IL-6 downregulation, while quercetin 4’-*O*-glucoside, isorhamnetin 4’-*O*-glucoside, and *p*-coumaroyl glycolic acid showed the highest correlation to IFN-*γ* and TNF-*α* inhibition.

**Conclusion:**

This study provides a fresh perspective on onion waste as a valuable source of bioactive constituents that could serve as the cornerstone for developing new, effective anti-PDE-5 and anti-inflammatory drug candidates.

**Supplementary Information:**

The online version contains supplementary material available at 10.1186/s12906-024-04559-2.

## Background

Onion (*Allium cepa* L.) is a widely distributed member of the *Allium* genus, ranking second in commercial importance after tomatoes. The plant comprises many cultivars, each has its own peculiar flavor, and they usually exist in various colors such as red, yellow, white, green, and purple [1, 2]. Onion represents a rich source of diverse phytochemicals with various beneficial health promoting activities such as organo-sulfur compounds, flavonoids, phenolic acids, saponins and fatty acids [3–5]. Since immemorial times, onion has been cultivated and utilized as a nutrient and has been traditionally used to help with inflammatory disorders and sexual impotence [4, 6, 7]. In ancient Egypt, people recognized onion as a plant with aphrodisiac activity and as a remedy for a number of inflammatory conditions [6]. In Eastern Nigeria and Uganda, people have used onion bulb extract to enhance low sperm count, improve erectile function and relieve testicular pains [7, 8]. Moreover, the fresh juice of the bulb was advocated in various countries for relieving pain and swelling [9]. Daily consumption of onion and processing by the food industry leads to the production of huge amounts of onion solid waste constituted mainly by outer dry papery scales and roots. Onion waste was reported to be rich in diverse phytochemicals with promising health functionalities, and hence the need for its valorization captured the concern of many scientists [10].

Male erectile dysfunction (ED) or impotence is defined as incompetence to accomplish, sustain, or maintain sufficient penile tumescence for satisfactory sexual performance. [11]. Persistent, low-grade inflammation significantly contributes to the onset of ED. Inflammation can lead to damage to the inner lining of blood vessels, a condition known as endothelial dysfunction, which can particularly impact the blood vessels and nerves crucial for erectile function [12, 13]. Metabolic disorders such as obesity, metabolic syndrome, and type 2 diabetes, which are linked with chronic low-grade inflammation, are seen as potential contributors to endothelial dysfunction and are acknowledged risk factors for sexual dysfunction [14]. Therefore, managing inflammation through lifestyle modifications and medication could potentially enhance erectile function.

Phosphodiesterase 5 (PDE-5), a predominant cyclic guanosine monophosphate (cGMP)-degrading enzyme, was found to be upregulated in penile corpus cavernosa tissues in ED patients. Hence, inhibiting such enzyme could be of pharmacological significance in ED treatment [15]. Currently, synthetic PDE-5 inhibitors including sildenafil citrate, tadalafil and vardenafil have become the first-line treatment for ED. Nonetheless, their use has been associated with several harmful effects [16]. Accordingly, the urge for screening new selective PDE-5 inhibitors - particularly from natural sources - is rising and captivating scientific attention.

In this study, the chemical profiles of peel and root extracts of four onion cultivars were investigated using UPLC-MS/MS analysis. The acquired data were further subjected to principal component analysis (PCA) to define both similarities and differences among different samples. Further, the extracts were biologically assessed for their PDE-5 inhibitory and anti-inflammatory activities. The results of biological activities were correlated to chemical profiles of onion waste samples using partial least squares (PLS) analysis to unravel putative biomarkers that are likely to mediate the tested activities, thereby allowing for their utilization as potentially useful constituents.

## Methods

### Reagents and apparatus

Please refer to supplementary material.

### Onion collection and sample preparation

Cultivated bulbs of four *Allium cepa* L. cultivars, red onion “Giza red”, yellow onion which is represented by two cultivars; the golden yellow “Giza 6 Mohassan” cultivar and the copper-yellow “Giza 20” cultivar in addition to white onion “Giza white”, were purchased from Agricultural Research Station Farm, Shandawil Village, Sohag Governorate in April 2022. The phenotypic characters of each cultivar are depicted in Table S1. The cultivars were identified and authenticated by Dr Refat Allam, Assistant Professor, Onion Research Dept., Crops Res. Institute, Agricultural Research Center (ARC), Shandawel Research Station, Sohag Governorate, and voucher specimens of the cultivars: red onion (ACR), yellow onion cultivars; (ACY20) & (ACY6M) and white onion (ACW), were deposited in the herbarium of faculty of pharmacy, Alexandria University for reference.

Each of these cultivars was cleaned and divided into husks (peels) and basal plates with attached roots (roots). Each sample was divided into three sets to obtain a total of 24 samples (Table S2). Samples were subjected to air-drying for a week. Dried samples were separately ground into a fine powder, extracted using aqueous ethanol (70% v/v), filtered, concentrated using a rotary evaporator and finally freeze-dried. Samples were stored at -20 °C until further analysis. Twenty mg from each of the onion samples was dissolved in 2 mL of UPLC grade methanol, sonicated for 5 min at 25 ◦C then filtered using disposable nylon filters (pore size 0.22 μm) to be used for both qualitative and semi-quantitative determination of compounds.

### Chemical profiling using UPLC–ESI–MS/MS

#### UPLC experiment conditions

The UPLC system encompassed a Waters Acquity QSM pump, an LC-2040 (Waters Corporation) autosampler, degasser and Waters Acquity CM detector. Samples were separated using a Waters Acquity UPLC BEH C18 column (1.7 μm particle size – 2.1 × 50 mm). A binary mobile phase was prepared by filtration using 0.2 μm filter comprising membrane disc and degassed by sonication before injection. The mobile phase consisted of water + 0.1% (v/v) formic acid (A) and methanol + 0.1% (v/v) formic acid (B). The mobile phase was pumped at 0.2 mL/min into the UPLC system with injection volume of 5 µL and programmed as gradient elution through 32 min performed as the following sequences: 0.0–2.0 min, 10% B; 2.0–5.0 min, 30% B; 5.0–15.0 min, 70% B; 15.0–22.0 min, phase 90% B; 22.0–25.0 min, 90% B; 26.0 min, 100% B; 26.0–29.0 min, 100% B; 29.0–30.0 min, 10% B.

#### ESI-MS conditions

For LC/MS analysis, a triple quadruple (QqQ) mass spectrometer was coupled to the UPLC instrument via an ESI interface. Ultra-high purity helium was used as the collision gas and high purity nitrogen as the nebulizing gas. The mass spectrometer was monitored in both negative and positive ionization modes over 50–1200 *m/z* mass range. The optimized detection parameters were as follows: temperature 150 °C, cone voltage 30 V, capillary voltage 3 kV, desolvation temperature 440 °C, cone gas flow 50 L/h, and desolvation gas flow 900 L/h. The analysis process run time lasted for 32 min. Regarding automatic MS/MS fragmentation process of the precursor ions that have been filtered by the first quadrupole (Q1), then in the second quadrupole (Q2) the mass fragmentation was performed through collision-induced dissociation (CID) energy that was ramped from 30 to 70 eV utilizing Ultra-high purity helium as collision gas. Eventually, the third quadrupole mass analyzer (Q3) filtered the daughter ions produced from CID that consequently related to the molecular structure of the precursor ions.

#### Annotation of metabolites

The raw UPLC–MS data were pre-processed using Mzmine^®^ version 2.8 software that has been utilized for importing data, peak deconvolution, alignment, and annotation. Base peak chromatograms (BPC) of the samples were presented in Figure S1. Metabolites were annotated via comparing their retention times relative to external standards, interpreting tandem mass spectra (quasi-molecular ions as well as diagnostic MS/MS fragmentation profiles) combined with our in-house comprehensive database that was set up covering all compounds previously reported in the literature in different onion cultivars including Dictionary of Natural Products (https://dnp.chemnetbase.com/*)*, PubChem (*PubChem (nih.gov)*) and Massbank (https://massbank.eu/MassBank/*)* to provide high confidence level of annotation (level II) [17]. The structures of some of the annotated compounds are provided in (Figure S2).

#### Semi-quantitation of metabolites using standard solutions

Standard compounds (alliin, ferulic acid, quercetin, *β*-chlorogenin, and palmitic acid) were used for the relative quantification of the identified metabolites (Table S3). The concentration of each metabolite was denoted in terms of milligrams of standard equivalents per gram of dry extract of each sample (Table S4). The relative concentrations of each class of metabolites in the tested samples are depicted in Figure S3. For detailed procedure, please refer to supplementary material.

### Evaluation of PDE-5 inhibitory activity

Please refer to supplementary material.

### Evaluation of cytotoxicity and anti-inflammatory activity

#### Human white blood cells isolation and cultivation

Human WBCs isolation and cultivation were done based on Mosmann, 1983 [18]. Cytotoxicity is a part of anti-inflammatory assay in order to assess the safety of tested extracts on WBCs. For details of the procedure, please refer to supplementary material.

#### Evaluation of cytotoxicity (MTT assay)

According to the MTT assay, metabolically active living cells were detected by mitochondrial succinate dehydrogenase that converts the MTT into a dark purple formazan. The quantitation of formazan was done spectrophotometrically. Cell cytotoxicity 50 (CC_50_) was calculated by the GraphPad software (GraphPad Software Inc, California) using the % viability calculated from the serial dilutions of the test sample. For details of the procedure, please refer to supplementary material.

#### Evaluation of the effective anti-inflammatory concentrations (EAICs) in LPS-stimulated WBCs

LPS acts as a common inflammatory inducer and causes abnormal up-regulation in the proliferation of human leukocytes. The abnormal increase in cell proliferation can be used as a marker of inflammation. This assay was performed according to Mosmann, 1983 [18]. EAICs of test samples were calculated using the GraphPad software. For details of the procedures, please refer to supplementary material.

#### Extraction of RNA of untreated and treated LPS-stimulated human WBCs and cDNA synthesis

Please refer to supplementary material.

#### Determination of the levels of pro-inflammatory markers (TNF-*α*, IFN-*γ*, IL-1*β* and IL-6)

Real time polymerase chain reaction (PCR) was utilized to determine the expression levels of [tumor necrosis factor-*α* (TNF-*α*), interleukin-1*β* (IL-1*β*), interferon-*γ* (IFN-*γ*) and IL-6] in lipopolysaccharide (LPS)-stimulated human WBC’s before and after treatment with different onion samples. Results were expressed as means ± standard deviation (SD) of three determinations. For details of the procedure, please refer to supplementary material.

### Determination of the PDE-5 inhibitory activity and anti-inflammatory biomarkers using PLSR analysis

In order to model the PDE-5 inhibitory and anti-inflammatory activities exerted by onion peel and root extracts, a partial least square regression (PLSR) model was created for each activity. The samples were divided into 2 sets: calibration (32 samples) and test ones (16 samples). The models generated using calibration samples were evaluated using R^2^ as a gauge of quality of fit, observed versus predicted plots, and root mean square error of calibration (RMSEC). To ensure the reliability and accuracy of the developed PLSR models, a Leave-One-Out (LOO) cross-validation strategy was implemented, and the root mean square error of cross-validation (RMSECV) was computed. Permutation plots were then utilized to validate the predictability of the models and prevent overfitting of the data. Observed versus predicted plot, permutation plot, and RMSEC for PDE-5 inhibitory activity model are shown in Figure S4, while the corresponding plots for the anti-inflammatory activity model are depicted in Figure S5. The test samples were used for external validation of the models, and root mean square error of prediction (RMSEP) was calculated. The performance parameters observed for both activity models fall within the acceptable ranges, with an R^2^ value exceeding 0.9 or approaching 1 which indicates data fitting (Table S5). The analysis of permutation plots indicated that the intercept values were below 0.6 which in turn reflects reliability and good predictability of the created models. Moreover, permutation plots ensured that constructed models were not overfitting the noise present in the data. Additionally, the models were evaluated in terms of classification and prediction parameters including sensitivity, specificity, accuracy, and efficacy (Tables S6 & S7).

### Multivariate data analysis

To outline similarities and differences between peel and root extracts of the four onion cultivars based on their chemical composition, Metaboanalyst 4.0 (http://www.metaboanalyst.ca/*)* was utilized to create hierarchical cluster analysis (HCA) heat maps. Unsupervised and supervised multivariate analysis of data; PCA, PLS, and PLS validation (internal and external validation) were accomplished utilizing SIMCA-P version 14.0 software (Umetrics, Sweden) to maximize the separation of samples, and explain the clustering pattern of onion samples in relation to their biological activities to pick out the biomarkers positively correlated to such activities.

### Statistical analysis

One-way variance of analysis (ANOVA) using the software SPSS 26.0 (SPSS Inc., Chicago, IL. USA) was used for the analysis of semi-quantitation results and biological activities. Results with a p-value of less than 0.01 were considered significant.

## Results

### Chemical profiling using UPLC–ESI–MS/MS

Reversed-phase UPLC–ESI–MS/MS using a triple quadruple (QqQ)–MS analyzer was employed for phytochemical analysis of peel and root extracts of the tested onion cultivars. The ionization of compounds was accomplished in both positive and negative electrospray ionization (ESI) modes to achieve efficient and sensitive detection of the compounds. Negative-ion MS spectra imparted superior sensitivity and more detectable peaks compared to positive-ion mode, particularly in the elution region of flavonoids and saponins. Moreover, it revealed more intense [M − H] ^−^ ions and lower noise giving rise to higher signal-to-noise ratios and better sensitivity. On the other hand, positive ion mode was more efficient for the analysis of amino acids and *γ*-glutamyl peptides [19, 20]. A total of **103** chromatographic peaks were retrieved from the examined samples comprising diverse phytochemical classes, e.g., amino acids, peptides, phenolic acids, flavonoids, saponins, and fatty acids. The identified compounds were presented in Table 1 along with their retention times (t_R_), chemical class, quasi-molecular ions, molecular formula, and the MS/MS ions used for the identification. BPC of peel and root extracts of the four cultivars were presented in Figure S1. The structures of the annotated compounds were previously published in a review article by Elattar et al., 2024 [21].

Standard compounds (alliin, ferulic acid, quercetin, *β*-chlorogenin and palmitic acid) were efficiently utilized for relative quantitation of all the identified metabolites including amino acids, peptides, phenolic acids, organic acids, flavonoids, saponins and fatty acids (Table S3). The concentration of each metabolite was expressed as mg standard equivalents per g dry extract of each tested sample (Table S4). Figure S3 presents the relative concentrations of each metabolite class in peel and root extracts of the four onion cultivars. Regarding peel extracts of the four cultivars, flavonoids were the most predominant class in red onion followed by saponins, peptides, phenolic acids, fatty acids and finally hydroxycinnamic acid amides (HCAAs). A similar pattern was observed for yellow onion cultivars, though they displayed higher concentrations of phenolic acids relative to peptides and dominancy of HCAAs over fatty acids. On the other hand, white onion peel extract exhibited a very different pattern where peptides and amino acids showed the highest concentrations followed by flavonoids. Additionally, it showed the lowest concentration of all metabolite classes. Regarding root extracts, the four cultivars showed the same pattern, which was like that of red onion peel with the exception that HCAAs were more abundant than fatty acids in these extracts. It was observed that the highest concentration of flavonoids was detected in peel and root extracts of red onion, respectively, in line with previous studies [1].

**Table 1:** The identified metabolites in peel and root extracts of the tested onion cultivars by UPLC-ESI- MS/MS in positive and negative ionization modes

ID	Name	t_*R*_ (min)	Ion type	Class	Mwt	Formula	MS^*n*^ Fragments
**1**	Alanine^*^	1.14	[M + H]	Amino acid	89.09	C_3_H_7_NO_2_	45, 44
**2**	Arginine^*^	1.21	[M + H]	Amino acid	174.12	C_6_H_14_N_4_O_3_	158, 157, 130, 116, 70, 60
**3**	Propiin	1.26	[M − H]	*S*-alk(en)ylcysteine sulfoxide	179.24	C_6_H_13_NO_3_S	178, 136, 91, 48.9
**4**	Methionine^*^	1.32	[M − H]	Amino acid	149.21	C_5_H_11_NO_2_S	115, 100, 46.9
**5**	Ornithine^*^	1.36	[M + H]	Amino acid	132.16	C_5_H_12_N_2_O_2_	116, 115, 70
**6**	γ-Glutamyl-methionine sulfoxide	1.42	[M − H]	Sulfur-containing peptide	294.33	C_10_H_18_N_2_O_6_S	275, 164, 128
**7**	γ -Glutamyl-glutamine	1.46	[M − H]	Peptide	275.26	C_10_H_17_N_3_O_6_	256,230, 145, 128
**8**	(Iso)alliin	1.51	[M + H]	*S*-alk(en)ylcysteine sulfoxide	177.22	C_6_H_11_NO_3_S	161, 137, 120
**9**	Oxalic acid	1.54	[M − H]	Organic acid	90.03	C_2_H_2_O_4_	71, 48.9
**10**	Allicin^*^	1.59	[M − H]	Thiosulfinate	162.28	C_6_H_10_OS_2_	120.9, 89, 48.9
**11**	Tartaric acid	1.62	[M − H]	Organic acid	150.09	C_4_H_6_O_6_	87
**12**	Caffeic acid^*^	1.64	[M − H]	Phenolic acid	180.16	C_9_H_8_O_4_	135.04, 134.04, 89
**13**	Caffeoylquinic acid	1.66	[M − H]	Phenolic acid	354.31	C_16_H_18_O_9_	191,179,173, 161, 135
**14**	γ -Glutamyl-*S*-(prop- 2-enyl) cysteine sulfoxide	1.68	[M + H]	Sulfur-containing peptide	306.34	C_11_H_18_N_2_O_6_S	266, 217, 186, 177, 154, 130
**15**	Feruloylquinic acid	1.69	[M − H]	Phenolic acid	368.34	C_17_H_20_O_9_	193,191, 134
**16**	Malic acid^*^	1.72	[M − H]	Organic acid	134.09	C_4_H_6_O_5_	117, 71
**17**	Lunularic acid	1.84	[M − H]	Phenolic acid	258.27	C_15_H_14_O_4_	213, 107, 106
**18**	Pro Betaine (*N, N*-Dimethyl-Proline)	1.93	[M − H]	Amino acid	144.1	C_7_H_14_NO_2_^+^	100, 86, 84
**19**	Glutamic acid^*^	1.96	[M − H]	Amino acid	147.13	C_5_H_9_NO_4_	128, 102
**20**	Pipecolic acid^*^	1.98	[M + H]	Amino acid	129.16	C_6_H_11_NO_2_	84
**21**	*S*-Methyl methionine	1.99	[M − H]	Amino acid	164.24	C_6_H_14_NO_2_S	103, 101, 61, 45
**22**	Citric acid	2.02	[M − H]	Organic acid	192.12	C₆H₈O₇	111, 85
**23**	Vanillic acid	2.09	[M − H]	Phenolic acid	168.15	C_8_H_8_O_4_	153,137, 123
**24**	Pyroglutamic acid^*^	2.18	[M + H]	Amino acid derivative	129.11	C_5_H_7_NO_3_	84, 56, 41
**25**	Rosmarinic acid^*^	2.52	[M − H]	Phenolic acid	360.31	C_18_H_16_O_8_	179, 161, 135
**26**	γ-Glutamyl-*S*-(2-carboxypropyl) cysteine-glycine	2.55	[M + H]	Sulfur-containing peptide	393.41	C_14_H_23_N_3_O_8_S	263, 87, 74
**27**	γ -Glutamyl-methionine	2.59	[M − H]	Sulfur-containing peptide	278.32	C_10_H_18_N_2_O_5_S	233, 259, 148, 128
**28**	Cyanidin 3-*O*-(malonyl-acetyl)-glucoside	2.74	[M − 2 H], 593 [M − 2 H − H_2_O]	Anthocyanin	577.11	C_26_H_25_O_15_^+^	447, 285, 241
**29**	Glycolic acid^*^	2.86	[M − H]	Organic acid	76.05	C₂H₄O₃	57, 47, 45
**30**	γ-Glutamyl-*S*-(Propyl) cysteine sulfoxide	3.01	[M − H]	Sulfur-containing peptide	308.11	C_11_H_20_N_2_O_6_S	178, 136, 91, 48.9
**31**	Succinic acid	3.12	[M + H]	Organic acid	118.09	C_4_H_6_O_4_	101, 73, 55
**32**	Ferulic acid^*^	3.45	[M − H]	Phenolic acid	194.18	C_10_H_10_O_4_	178.4; 149.3, 134
**33**	*S*-(prop-1-enyl)cysteine sulfoxide-*S*-(prop-1-enyl) cysteine sulfoxide	3.58	[M − H]	Sulfur-containing peptide	336.09	C_12_H_20_N_2_O_5_S_2_	132, 74, 41
**34**	Ascorbic acid	3.75	[M − H]	Organic acid	176.12	C_6_H_8_O_6_	157, 115
**35**	Tryptophan^*^	4.07	[M + H]	Amino acid	204.23	C_11_H_12_N_2_O_2_	188, 146, 144, 118, 91
**36**	Sinapic acid^*^	4.17	[M − H]	Phenolic acid	224.21	C_11_H_12_O_5_	205, 179, 163
**37**	*p*-Coumaroyl glycolic acid	4.52	[M + H]	Phenolic acid	222.19	C_11_H_10_O_5_	205, 147, 119
**38**	Hydroxytyrosol	4.71	[M − H]	Phenolic compound	154.16	C_8_H_10_O_3_	135, 109
**39**	Asparagine^*^	5.29	[M − H]	Amino acid	132.12	C_4_H_8_N_2_O_3_	114, 113, 95, 70, 42, 58
**40**	Trihydroxyphenylglyoxylate	5.36	[M − H]	Phenolic compound	198.13	C_8_H_6_O_6_	179, 153
**41**	3’-Methoxylunularic acid	5.38	[M − H]	Stilbenoid (phenolic acid)	288.09	C_16_H_16_O_5_	243.1, 228, 150, 136
**42**	Lunularin 4-*O*-hexoside	5.44	[M − H]	Stilbenoid glycoside	376.15	C_20_H_24_O_7_	269, 213
**43**	Epigallocatechin^*^	5.61	[M − H]	Flavonoid	306.27	C_15_H_14_O_7_	261, 221, 219, 179 ,167, 165
**44**	Methoxytyramine	5.66	[M − H]	Amine	167.21	C_9_H_13_NO_2_	150, 122, 92
**45**	Apigenin *O*-pentosyl-hexoside	6.2	[M − H]	Flavonoid	564.49	C_26_H_28_O_14_	515, 269, 149, 131
**46**	γ -Glutamyl *S*-(prop-1-enyl) cysteine	6.78	[M − H]	Sulfur-containing peptide	290.1	C_11_H_18_N_2_O_5_S	245, 130, 73
**47**	Cyanidin 3-*O*-acetyl glucoside	7.58	[M − 2 H], [M − 2 H + H_2_O]	Anthocyanin	491.4	C_23_H_23_O_12+_	447, 285, 241
**48**	Cyanidin 3-*O*-malonylglucoside	7.72	[M − 2 H], [M − 2 H + H_2_O]	Anthocyanin	535.43	C_24_H_23_O_14_	447, 285, 241
**49**	Tropeoside B	7.79	[M − H]	Saponin	726.9	C_38_H_62_O_13_	593, 447, 429
**50**	Peonidin 3-*O*-glucoside^*^	8.02	[M − 2 H], [M − 2 H + H_2_O]	Anthocyanin	463.41	C_22_H_23_O_11_^+^	299, 285
**51**	Quercetin 3,4’-*O*-diglucoside^*^	8.13	[M − H]	Flavonoid	626.15	C_27_H_30_O_17_	463, 445, 301, 179, 161
**52**	Isorhamnetin 3,4’-*O*-diglucoside	8.38	[M − H]	Flavonoid	640.54	C_28_H_32_O_17_	477, 459, 315, 179, 161
**53**	2-(3,4-Dihydroxybenzoyl)-2,4,6- trihydroxy-3(2 H)-benzofuranone	8.66	[M − H]	Hydroxybenzoic acid derivative	318.03	C_15_H_10_O_8_	299, 273, 207, 191
**54**	Ascalonicoside A	8.91	[M − H]	Saponin	918.48	C_45_H_74_O_19_	771.4, 609.3, 447.3, 1
**55**	Dihydroquercetin 3-*O*-rhamnoside	9.04	[M + H]	Flavonoid	450.4	C_21_H_22_O_11_	303, 285, 151, 109
**56**	Kaempferol *O*-rhamnosyl-hexoside	9.16	[M − H]	Flavonoid	594.52	C_27_H_30_O_15_	575, 447, 285, 151
**57**	Ceposide A	9.21	[M − H + HCOOH]	Saponin	888.46	C_44_H_72_O_18_	741.4, 579.3, 447.3, 429.3
**58**	Quercetin 3-*O*-glucoside^*^	9.27	[M + H]	Flavonoid	464.38	C_21_H_20_O_12_	447, 303, 153, 109
**59**	Quercetin 4’-*O*-glucoside^*^	9.58	[M − H]	Flavonoid	464.38	C_21_H_20_O_12_	301, 179, 151
**60**	Ceposide C	9.69	[M − H]	Saponin	918.48	C_45_H_74_O_19_	771.4, 609.3, 447.3, 429.3
**61**	Isorhamnetin 3-*O*-glucoside^*^	9.71	[M − H]	Flavonoid	478.4	C_22_H_22_O_12_	315, 299, 151, 137
**62**	Isorhamnetin 4’-*O*-glucoside	10.05	[M + H]	Flavonoid	478.1	C_22_H_22_O_12_	317, 301, 153, 137
**63**	Adduct of quercetin with protocatechuic acid	11.49	[M − H]	Flavonoid	454.34	C_22_H_14_O_11_	437, 301
**64**	Quercetin dimer hexoside	10.89	[M − H]	Flavonoid	764.11	C_36_H_28_O_19_	611, 601, 600, 299
**65**	*N*-(*p*-Coumaroyl)-tyramine	10.97	[M + H]	Hydroxycinnamic acid amide	283.32	C_17_H_17_NO_3_	147, 138, 121
**66**	Hesperetin^*^	11.13	[M − H]	Flavonoid	302.28	C_16_H_14_O_6_	271, 151, 125
**67**	*N*-Feruloyl-tyramine	11.17	[M + H]	Hydroxycinnamic acid amide	313.12	C_18_H_19_NO_4_	177, 138, 121
**68**	Ceparocide I	11.21	[M − H]	Saponins	870.43	C_44_H_70_O_17_	723.5, 591.5, 573.5
**69**	Allylmercaptoglutathione	11.41	[M − H]	Sulfur-containing peptide	379.5	C_13_H_21_N_3_O_6_S_2_	249, 175, 146, 128
**70**	*N*-Feruloyl-3-methoxytyramine	11.41	[M + H]	Hydroxycinnamic acid amide	343.13	C_19_H_21_NO_5_	177, 168
**71**	Adduct of quercetin with methyl protocatechuate	10.45	[M − H]	Flavonoid	468.06	C_23_H_16_O_11_	453, 437, 301
**72**	Morin	11.6	[M − H]	Flavonoid	302.24	C_15_H_10_O_7_	285, 151, 136
**73**	Quercetin^*^	11.81	[M − H]	Flavonoid	302.03	C_15_H_10_O_7_	271, 179, 151
**74**	Tropeoside A methyl derivative	12.13	[M − H]	Saponin	770.43	C_40_H_66_O_14_	607.5, 475.5, 443.5
**75**	3-(Quercetin-8-yl)-2,3-epoxyflavanone	12.21	[M − H]	Flavonoid	602.47	C_30_H_18_O_14_	299,449
**76**	Taxifolin^*^	12.45	[M − H]	Flavonoid	304.25	C_15_H_12_O_7_	285, 275, 241, 177, 151, 125
**77**	3’-Hydroxymelanettin	12.63	[M − H]	Flavonoid	300.26	C_16_H_12_O_6_	299, 284, 256, 240
**78**	Dihydroxypegnadienone *O*- *O*-rhamnosyl-pentoside	12.68	[M − H]	Saponin	608.29	C_32_H_48_O_11_	461, 329, 311
**79**	Quercetin dimer hexoside isomer	12.75	[M − H]	Flavonoid	764.11	C_36_H_38_O_19_	611, 601, 600, 299
**80**	Sativanone	12.98	[M − H]	Flavonoid	300.31	C_17_H_16_O_5_	283, 241, 161, 137
**81**	Kaempferol^*^	13.2	[M − H]	Flavonoid	286.24	C_15_H_10_O_6_	243, 151, 133
**82**	Trihydroxyspirosten *O*- rhamnosyl-pentoside	13.23	[M − H]	Saponin	724.84	C_38_H_60_O_13_	578, 446, 428
**83**	Isorhamnetin^*^	13.42	[M − H]	Flavonoid	316.26	C_16_H_12_O_7_	273, 257 ,151, 135
**84**	Phloroglucinoyl dihydroxybenzoate	13.61	[M + H]	Dihydroxybenzoic acid derivative	262.22	C_13_H_10_O_6_	155, 127, 111
**85**	Randiasaponin IV	13.84	[M − H]	Saponin	913.09	C_47_H_76_O_17_	750, 604, 472, 454, 428
**86**	Quercetin dimer	14.3	[M − H]	Flavonoid	602.47	C_30_H_18_O_14_	449, 299
**87**	Trihydroxy- methoxyisoflavanone	14.57	[M + H]	Flavonoid	302.28	C_16_H_14_O_6_	27, 179, 151, 137
**88**	Adduct of quercetin dimer with methyl protocatechuate	14.89	[M − H]	Flavonoid	768.08	C_38_H_24_O_18_	611, 600, 299
**89**	Quercetin trimer	15.37	[M − H]	Flavonoid	902.08	C_45_H_26_O_21_	601, 599, 449, 299
**90**	Dihydroxy-dimethoxyisoflavone	15.49	[M − H]	Flavonoid	314.29	C_17_H_14_O_6_	285, 269, 161
**91**	Alliospiroside D	15.69	[M − H + HCOOH]	Saponin	754.9	C_39_H_62_O_14_	607, 445, 427
**92**	Alliospiroside B	16.01	[M − H]	Saponin	738.9	C_39_H_62_O_13_	591, 429, 411, 395
**93**	Naringenin^*^	17.64	[M − H]	Flavonoid	272.25	C_15_H_12_O_5_	150 118, 106
**94**	Alliospiroside A	17.64	[M – H + HCOOH]	Saponin	708.88	C_38_H_60_O_12_	561, 429, 411, 395
**95**	Oxo-octadecenoic acid	21.74	[M − H]	Fatty acid	296.44	C_18_H_32_O_3_	277, 251, 239
**96**	Myristic acid^*^	22.24	[M + H]	Fatty acid	228.37	C_14_H_28_O_2_	211, 193, 185, 62
**97**	Palmitic acid	22.39	[M + H]	Fatty acid	256.42	C_16_H_32_O_2_	239, 221, 213, 62
**98**	Stearic acid^*^	22.53	[M + H]	Fatty acid	284.48	C_18_H_36_O_2_	267, 249, 241, 56
**99**	*β*-Chlorogenin	23.08	[M − H]	Sapogenin	432.65	C_27_H_44_O_4_	413, 389, 359, 317
**100**	Oleic acid^*^	25.19	[M + H]	Fatty acid	282.46	C_18_H_34_O_2_	265, 241, 239, 56
**101**	Gadooleic acid	25.43	[M − H]	Fatty acid	310.52	C_20_H_38_O_2_	291, 265, 56
**102**	Linolenic acid	25.54	[M + H]	Fatty acid	278.43	C_18_H_30_O_2_	261, 243, 235, 233, 219, 56
**103**	Diosgenin^*^	27.63	[M − H]	Sapogenin	414.62	C_27_H_42_O_3_	395, 371, 341, 299

^*^ Denotes compounds identified by external standards

### Multivariate data analysis

#### Unsupervised pattern using heat map analysis

**Fig. 1:**
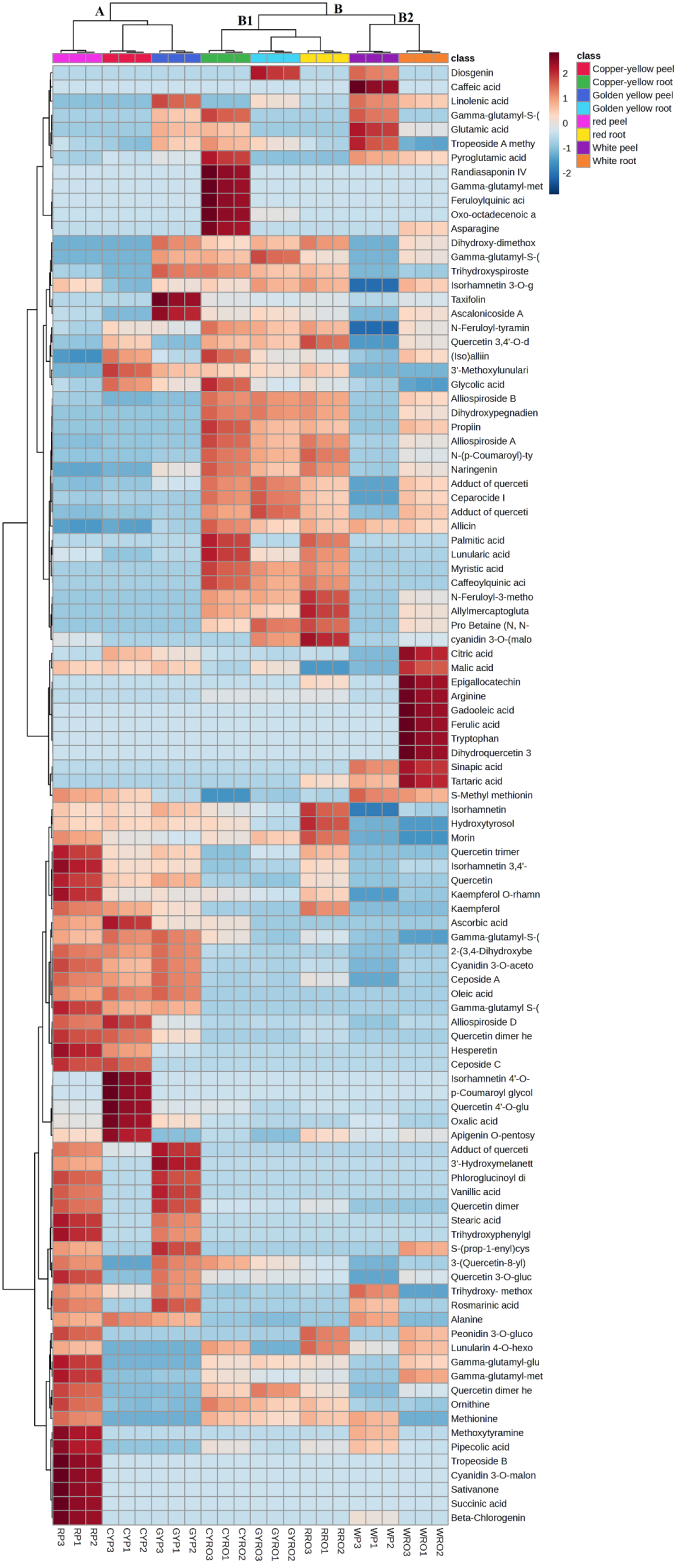
Hierarchical analysis heat map of all identified constituents in peel and root extracts of the tested onion cultivars. Brick red and blue indicate higher and lower abundances, respectively

UPLC-MS/MS data from peel and root samples of the four onion cultivars were processed by MetaboAnalyst 5.0 and subjected to HCA heat map analysis (Fig. 1). The HCA heat-map clearly showed two major clusters of three and five samples, denoted as groups A and B, respectively. Investigation of group A showed that peel samples of the three colored cultivars (red onion and the two yellow onion cultivars) clustered together which means that they were more closely related in their chemical profiles. Cluster B included root samples of the four cultivars together with white onion peel. These two main clusters were separately sub-clustered based on their chemical profiles’ likeness. Root samples of the three colored cultivars gathered in subgroup B1, while root and peel samples of white onion clustered together in subgroup B2.

Red onion peel was found to be abundant with the metabolites cyanidin 3-*O*-malonyl glucoside, quercetin, isorhamnetin 3,4’-*O*-diglucoside, quercetin trimer and tropeoside B. Meanwhile, copper-yellow peel showed predominance of quercetin 4’-*O*-glucoside, isorhamnetin 4’-*O*-glucoside, apigenin *O*-pentosyl-hexoside and ascorbic acid. Regarding golden yellow peel samples, they were rich in taxifolin, quercetin adduct with protocatechuic acid, 3’-hydroxymelanettin and ascalonicoside A. On the other hand, white onion peel demonstrated raised content of glutamic and caffeic acids. With respect to red onion root extracts, they showed the highest relative content of cyanidin 3-*O*-(malonyl-acetyl)-glucoside, *N*-feruloyl-3-methoytyramine and allylmercaptoglutathione. Moreover, copper-yellow root showed high abundance of feruloylquinic acid, randiasaponin IV and oxo-octadecanoic acid whereas diosgenin and γ-glutamyl-*S*-(prop-2-enyl) cysteine sulfoxide were abundant in golden yellow root. As for white onion root, ferulic acid, citric acid and tryptophan exhibited the highest abundance. It was evident that for each of the studied cultivars, peel extract displayed a higher abundance of flavonoids than root extract except for white onion. On the other hand, root extract was rich in saponins, HCAAs and fatty acids.

#### Unsupervised pattern recognition analysis using PCA

**Fig. 2:**
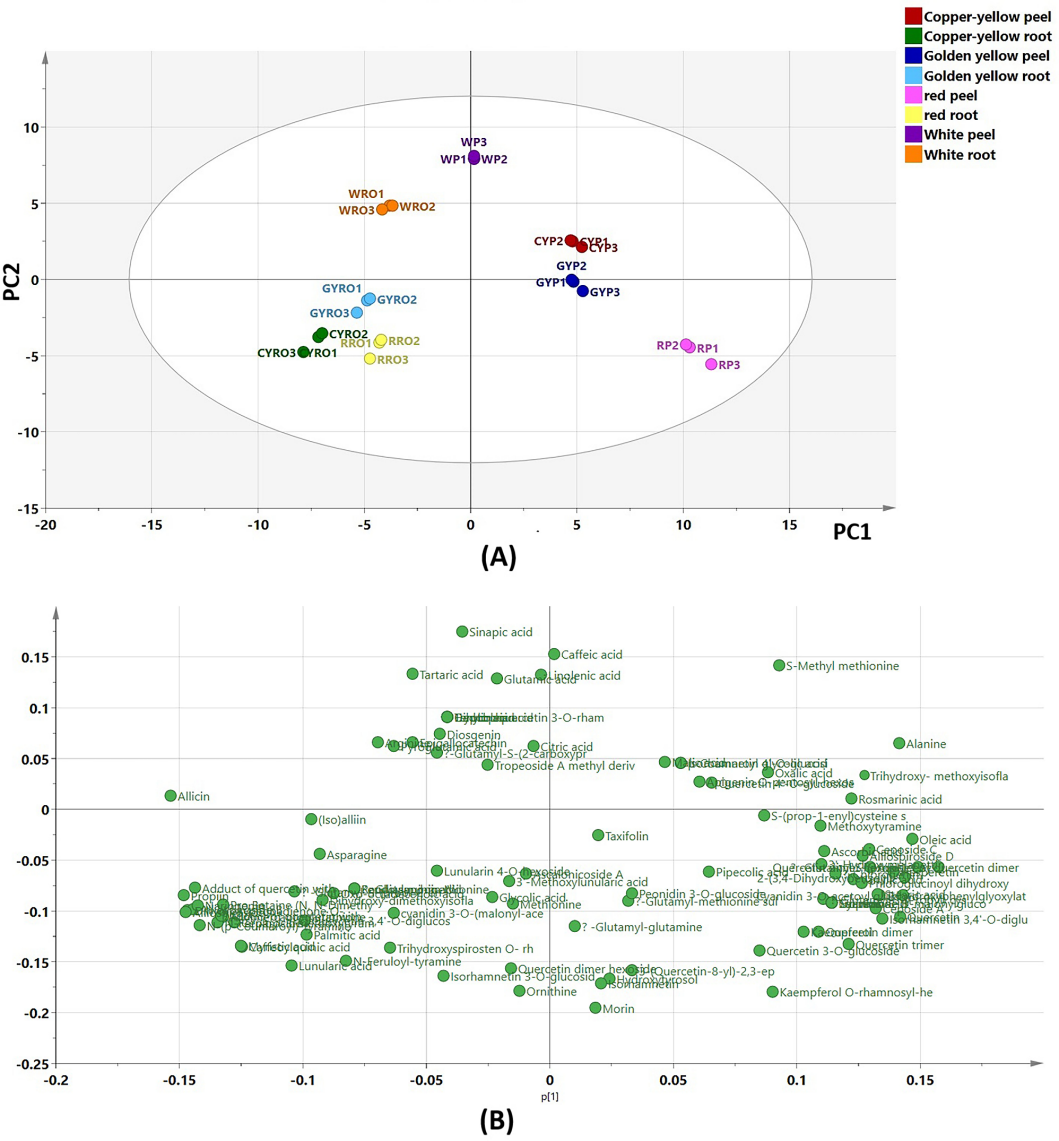
Principal component analysis (PCA) score scatter plot (**A**) and score loading plot (**B**) of peel and root samples of the four onion cultivars (*RP* Red peel, *WP* White peel *CYP* Copper-yellow peel, *GYP* Golden yellow peel, *RRO* Red root, *WRO* White root, *CYRO* Copper-yellow root, *GYRO* Golden yellow root)

The UPLC-MS/MS dataset was subjected to PCA as an unsupervised pattern recognition technique to reveal the clustering pattern among the different samples. The first two principal components represented 54.3% of total variation among samples. The PCA model was valid as inferred from the value of the goodness of fit (R^2^ = 0.998) and cross validation redundancy value (Q^2^ = 0.992) which reflected the reliability and the good predictability of the constructed model, respectively. The clustering pattern of the tested samples created by this model was consistent with that obtained from HCA analysis heat map (Fig. 2A). Peel samples of the tested cultivars were positioned along the positive side of PC1, while root samples were gathered along the negative side of the same principal component. Additionally, peel samples of white and copper-yellow onions, as well as root samples from white onions, were placed along positive side of PC2. Meanwhile, peel samples of red and golden yellow onions together with root samples of the three colored cultivars were located along the negative side of the same principal component. Moreover, it was revealed that peel samples of yellow onion cultivars grouped together whereas peel samples of white onion root were placed very close to the PC1 separating it from root samples.

The loading plots (Fig. 2B) were also created to unveil the metabolites that are likely to mediate the clustering of the different samples. Loading plots are tools that track the covariance between variables. They can be utilized to decipher patterns seen in the score plot, scores, and loadings [22]. The compounds responsible for the grouping of yellow peel samples were identified as quercetin 4’-*O*-glucoside, isorhamnetin 4’-*O*-glucoside, apigenin *O*-pentosyl-hexoside, and malic acid. On the other hand, the compounds associated with the clustering of red peel samples were quercetin, isorhamnetin 3,4’-*O*-diglucoside, cyanidin 3-*O*-acetyl-glucoside, phloroglucinoyl dihydroxybenzoate, vanillic acid, hesperetin, ceposide A, 2-(3,4-dihydroxybenzoyl)-2,4,6-trihydroxy-3(2 H)-benzofuranone and trihydroxy-phenylglyoxylate. The metabolites responsible for the clustering of white peel samples were identified as caffeic and linolenic acids. In addition, the compounds associated with the grouping of white root samples were identified as pyroglutamic, gadoleic acids, diosgenin, epigallocatechin, arginine, and *γ*-glutamyl-*S*-(2-carboxypropyl) cysteine-glycine. Lastly, the metabolites associated with the close placement of colored root samples were identified as asparagine, (iso)alliin, oxo-octadecenoic acid, dihydroxy-dimethoxyisoflavone, isorhamnetin 3,4’-*O*-diglucoside, randiasaponin IV, palmitic acid, and cyanidin 3-*O*-(malonyl-acetyl)-glucoside.

### Evaluation of PDE-5 inhibitory activity

All the tested onion peel and root extracts (25 µg/mL**)** significantly inhibited PDE-5 enzyme compared to the negative control showing varying degrees of potency (Table 2). The negative control for this assay was the reaction mixture prepared by adding 5 mM of the substrate (*p*-nitrophenyl phenylphosphate), 100 µL of the phosphodiesterase enzyme, and 20 mM Tris-HCl buffer (pH 8.0). This mixture demonstrated 100% enzyme activity. Of all extracts tested, root extract of golden yellow onion exerted a significant potent PDE-5 inhibitory activity showing IC_50_ of (7.86 ± 0.011 µg/mL) which was comparable to that of the standard, sildenafil (IC_50_ 6.25 ± 0.01 µg/mL). Among peel samples, peel extracts of copper-yellow and red onions exhibited significant PDE-5 inhibitory potentials with very close IC_50_ values (10.73 ± 0.021 and 10.76 ± 0.15 µg/mL), respectively (Table 2).

**Table 2:** % PDE activity at extract conc. (25 µg/mL), IC_50_ for PDE-5 inhibitory activity (µg/mL), effective anti-inflammatory concentrations (EAICs in µg/mL) and cytotoxicity CC_50_ (µg/mL) displayed by peel and root extracts of the tested onion cultivars and standard drugs

		^**^PDE-5 inhibitory activity		^**^Anti-inflammatory activity	
	Sample	%PDE enzyme activity at extract conc. (25 µg/mL)	IC _50_ (µg/mL)	Cytotoxicity CC_50_ (µg/mL)	EAIC (µg/mL)
Peel samples	Red onion	^*^ 20.2 ± 0.015	10.78 ± 0.15	704 ± 3.46	14.51 ± 0.018
	Copper-yellow onion	^*^ 12.2 ± 0.035	10.73 ± 0.021	414 ± 2.52	8.71 ± 0.01
	Golden yellow onion	^*^ 51.1 ± 0.32	23.04 ± 0.019	512 ± 3.84	10.77 ± 0.022
	White onion	^*^ 26.2 ± 0.04	13.29 ± 0.013	540 ± 2.71	11.36 ± 0.015
Root samples	Red onion	^*^ 10 ± 0.021	9.26 ± 0.018	773 ± 2.92	16.10 ± 0.027
	Copper-yellow onion	^*^ 35.6 ± 0.23	17.87 ± 0.024	854 ± 3.54	18.36 ± 0.014
	Golden yellow onion	^*^ 7.1 ± 0.011	7.86 ± 0.011	950 ± 3.86	21.55 ± 0.025
	White onion	^*^ 25.3 ± 0.02	14.91 ± 0.013	1005 ± 4.2	59.13 ± 0.26
	Sildenafil	^*^ 4 ± 0.021	6.25 ± 0.01		
	Negative control	^*^ 100 ± 0.88			
	Piroxicam			120 ± 1.9	30 ± 0.021

^*^Denotes that results are significant with *p-*value less than 0.01

^**^Results are the average of three determinations ± SD

### Evaluation of cytotoxicity and anti-inflammatory activity

The safety of the extracts was investigated using MTT cytotoxicity test, where CC_50_ values were determined for the tested samples as well as the standard piroxicam. Piroxicam is a potent non-steroidal anti-inflammatory drug that is typically utilized as a reference medication to evaluate and compare the anti-inflammatory properties of extracts and even new entities [23]. As depicted in Table 2, all tested extracts exhibited superior safety profile compared to piroxicam (CC_50_ value of 120 ± 1.9 µg/mL). Root extracts of the four cultivars exhibited higher safety profiles compared to peel extracts showing CC_50_ values between 773 and 1005 µg/mL. However, the calculated CC_50_ values for peel extracts ranged from 414 to 704 µg/mL.

All the tested extracts exerted significant anti-inflammatory activity in LPS-stimulated WBCs showing variable degrees of efficacy (Table 2). The tested extracts displayed EAICs ranging from 8.71 to 59.13 µg/mL which were lower than that of piroxicam (30 ± 0.021 µg/mL) except for white onion root extract since it showed EAIC of (59.13 ± 0.26 µg/mL) but was still effective. Peel extracts exhibited higher anti-inflammatory activities than those displayed by root extracts as inferred from their lower EAIC values, where the most promising anti-inflammatory activity was exerted by peel extract of copper-yellow onion (EAIC = 8.71 ± 0.01 µg/mL). Regarding root samples, root extract of red onion was the most active one (EAIC = 16.10 ± 0.027 µg/mL).

**Fig. 3:**
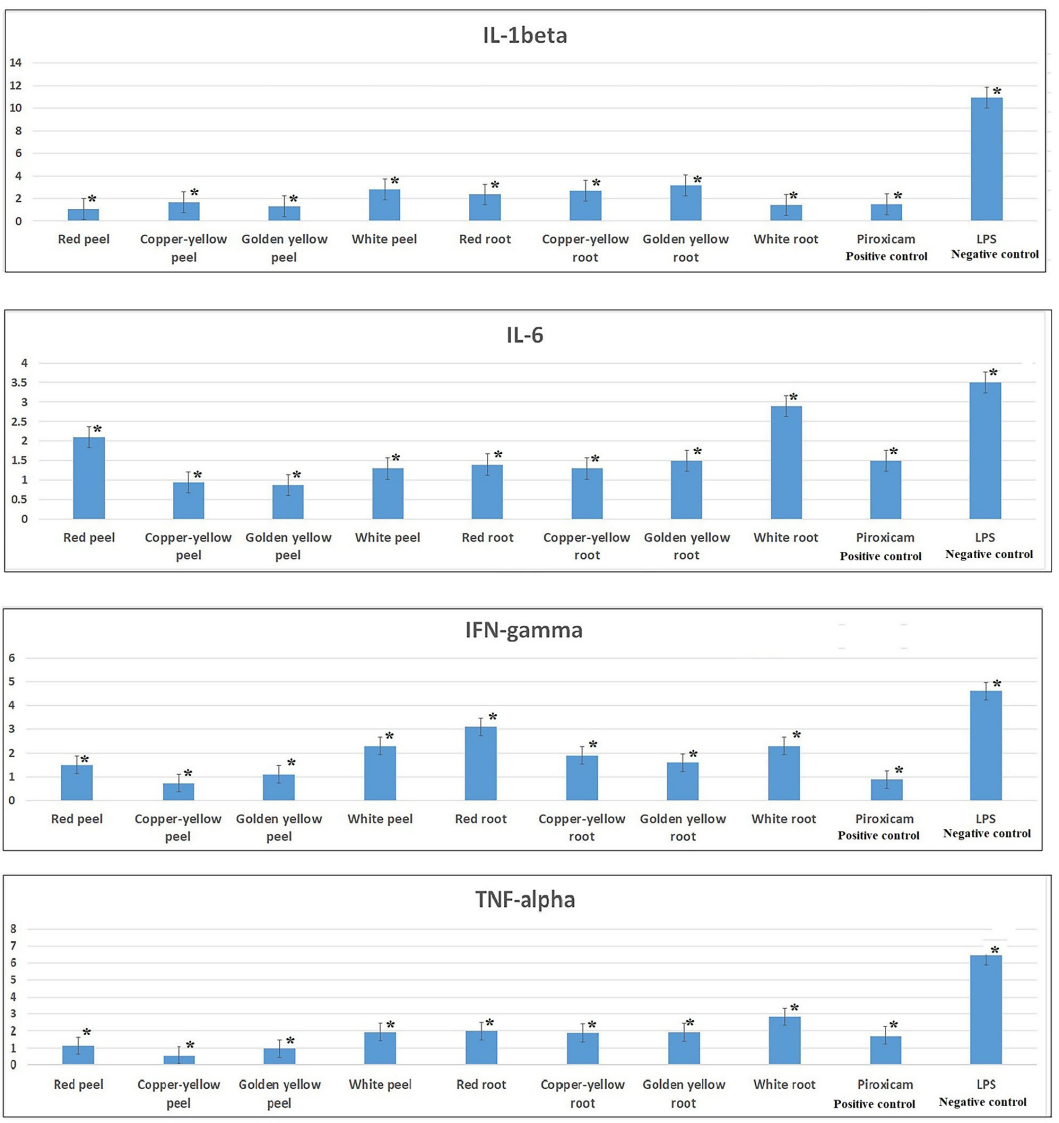
Bar charts demonstrating the levels of TNF-*α*, IL-1*β*, IFN-*γ* and IL-6 (expressed as fold change) in LPS-stimulated WBCs before and after treatment with peel, root extracts of the tested onion cultivars and piroxicam. *Denotes statistically significant results with *p-*value < 0.01

The mechanism underlying the promising anti-inflammatory activity exerted by onion waste extracts was explored via determining the gene expression of four pro-inflammatory markers (TNF-*α*, IL-1*β*, IFN-*γ* and IL-6) in LPS-stimulated WBCs before and after treatment with onion extracts. In this study, the negative control was represented by LPS-stimulated WBCs. All the tested samples significantly decreased the gene upregulation of the four proinflammatory cytokines in LPS-treated WBCs exhibiting varying degrees of potency. Peel extract of copper-yellow onion exerted the most potent anti-inflammatory activity regarding suppressing the upregulation of TNF-*α* and IFN-*γ* genes since the upregulation of the two genes declined to be 0.55-fold and 0.73-fold, respectively which was significantly lower than that shown by piroxicam (1.73 and 0.89-fold, respectively) (Fig. 3**)**. As for IL-1*β* and IL-6 genes, peel extracts of red and golden yellow onions were the most effective downregulators of these two pro-inflammatory cytokines by 0.88 and 1.05-folds, respectively. Interestingly, both extracts exhibited greater efficacy compared to piroxicam (Fig. 3**)**.

### Determination of the PDE-5 inhibitory activity and anti-inflammatory biomarkers using PLSR analysis

#### Determination of the PDE-5 inhibitory activity biomarkers

To investigate the subsequent grouping of the tested extracts in relation to their PDE-5 inhibitory activity, as well as to determine the biomarkers mediating such activity, UPLC-MS/MS data matrix (X-variables) and IC_50_ values (Y-variables) were imported for PLS model construction. PLS is a statistical technique that identifies relationships between two matrices (X and Y) by modeling their covariance structures. It proves to be especially beneficial when dealing with a large number of predictor variables that are highly correlated or collinear. PLS forms new predictor variables, known as components, as linear combinations of the original predictors, which are structured to have maximal covariance. PLS stands as a powerful tool in correlation analysis, attributed to its capacity to manage multicollinearity, accommodate a vast array of predictors, and its robustness in prediction. It not only measures the correlation but also predicts one variable based on the other, marking its worth in this field [24, 25].

**Fig. 4:**
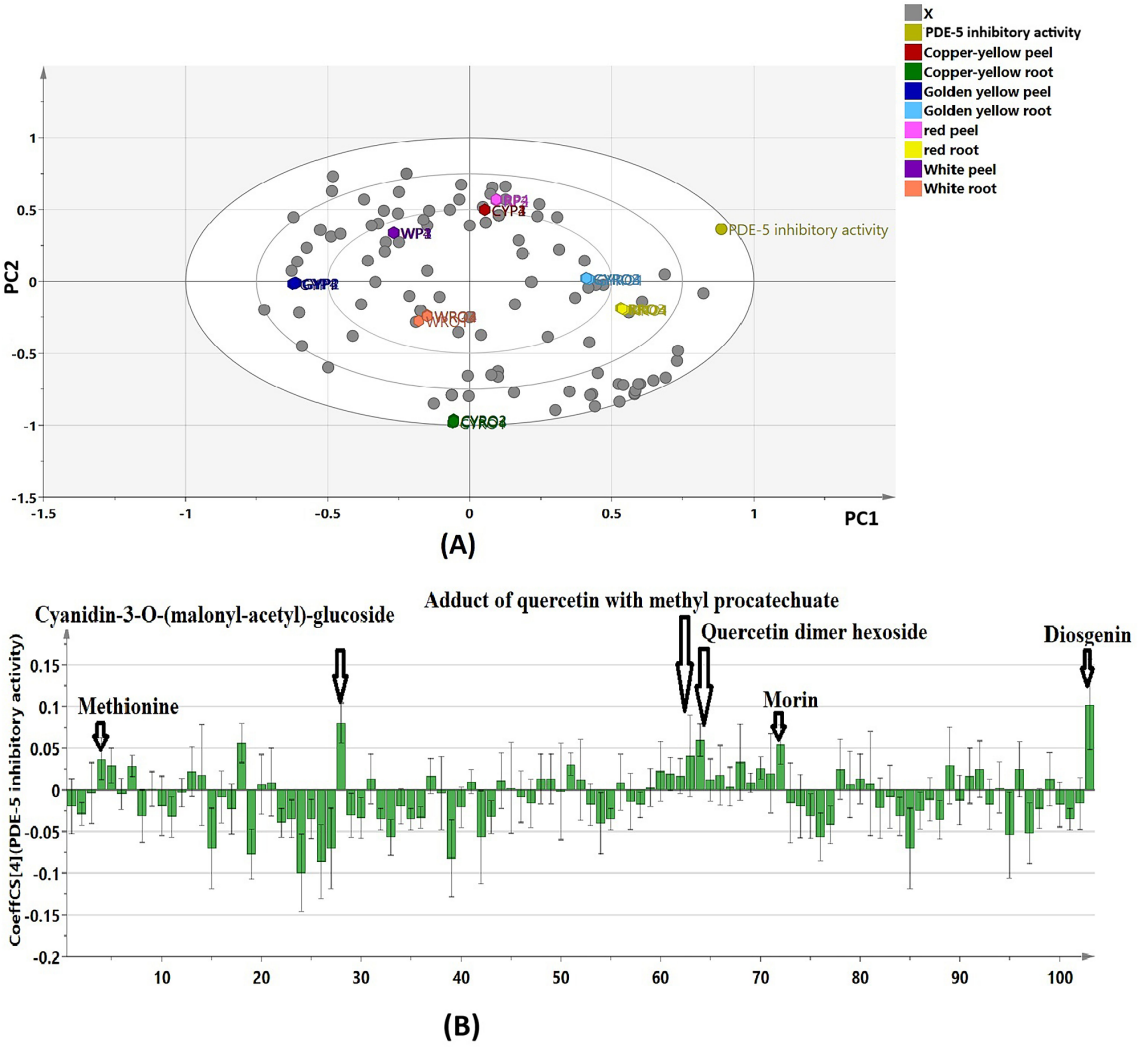
**A** Biplot of PLS model of peel and root samples of the tested onion cultivars in correlation to their PDE-5 inhibitory activity (*RP* Red peel, *WP* White peel, *CYP* Copper-yellow peel, *GYP*,Golden yellow peel, *RRO*, Red root, *WRO* White root, *CYRO* Copper-yellow root, *GYRO* Golden yellow root). **B** Coefficient plots of PLS model to show metabolites correlated to PDE-5 inhibitory activity displayed by peel and root extracts of the four onion cultivars

The constructed model showed accepted degrees of stability and reliability as manifested by the determined values for goodness of fit (R^2^ = 0.992) and predictability (Q^2^ = 0.982). The biplot (Fig. 4A) showed a strong spatial correlation between PDE-5 inhibitory activity and root samples of golden yellow and red onions, as these extracts demonstrated the highest levels of activity. Additionally, there was a less pronounced correlation observed with the peel samples of red and copper yellow onions. On the other hand, root samples of white and copper-yellow onions together with peel samples of white and golden yellow onions were positioned on the far-left side with negative PC1 values away from PDE-5 inhibitory activity as they showed lower efficacy.

The coefficient plot (Fig. 4B) was constructed to unravel the metabolites positively correlated to the PDE-5 inhibitory potential of onion waste extracts. Six compounds possessing a positive contribution to the PDE-5 inhibitory activity were identified. These compounds were among the top VIP metabolites; cyanidin 3-*O*-(malonyl-acetyl)-glucoside (VIP value = 2.18), quercetin adduct with methyl protocatechuate (VIP value = 1.21), methionine (VIP value = 1.15), morin (VIP value = 1.69), quercetin dimer hexoside (VIP value = 1.51) and diosgenin (VIP value = 1.7). Such metabolites exhibited high abundance in the samples with the highest activity.

#### Determination of the anti-inflammatory biomarkers

**Fig. 5:**
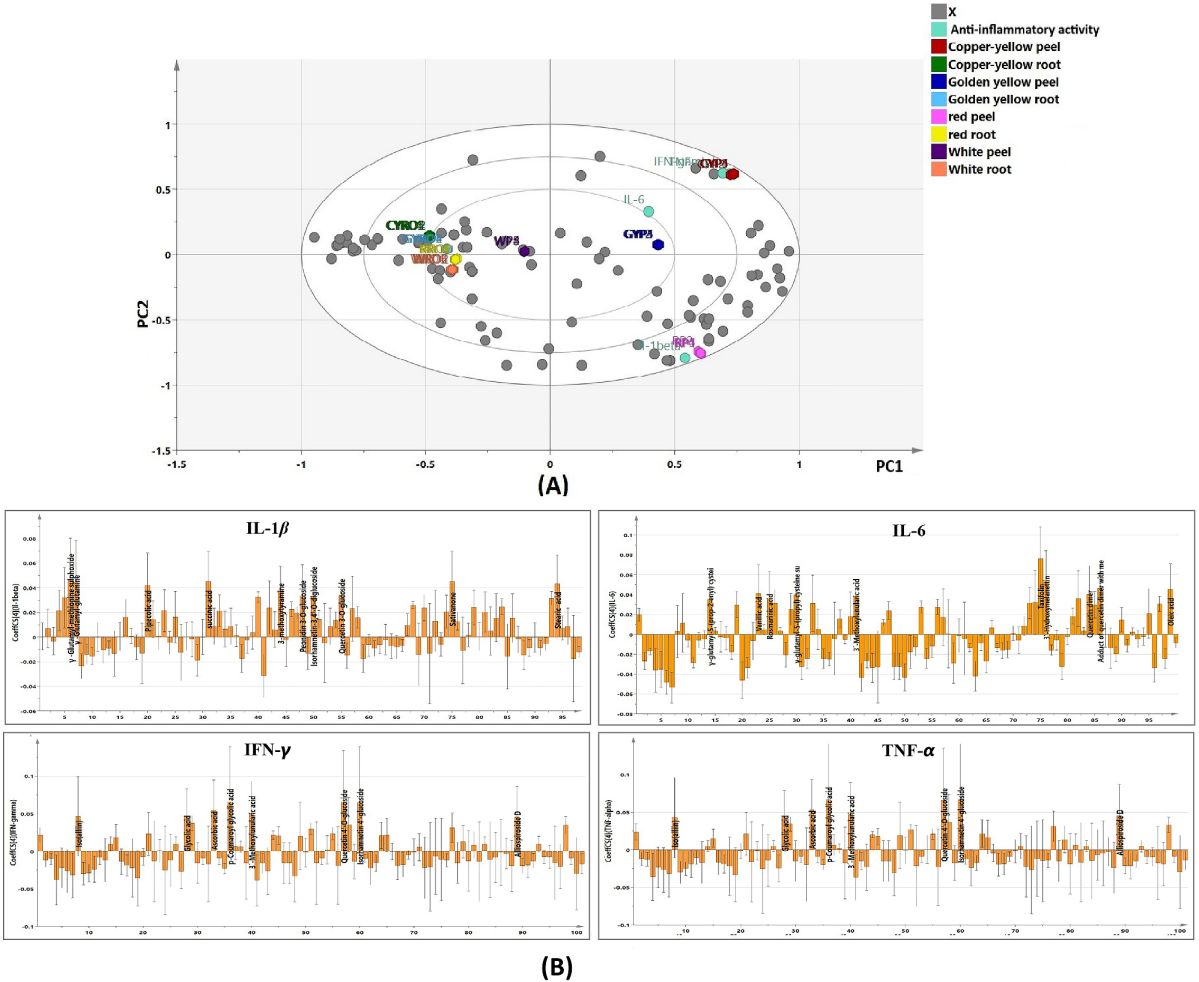
**A** Biplot of PLS model of peel and root samples of the tested onion cultivars in correlation to the proinflammatory markers inhibition levels (*RP* Red peel, *WP* White peel, *CYP* Copper-yellow peel, *GYP* Golden yellow peel, *RRO* Red root, *WRO* White root, *CYRO* Copper-yellow root, *GYRO*, Golden yellow root). **B** Coefficient plot of PLS model to show metabolites correlated to the proinflammatory markers inhibition levels in root and peel extracts of the four onion cultivars

To investigate the clustering of onion samples in terms of their anti-inflammatory potential as well as define the bioactive metabolites that are positively correlated to such activity, a PLS model was created. The obtained findings revealed the good predictive ability and goodness of fit of the constructed PLS model as inferred from the determined R^2^ (0.975) and Q^2^ (0.961) values. By referring to Fig. 5A, the biplot showed that peel samples of red, golden yellow and copper-yellow onions were positioned along the positive side of PC1 displaying a strong spatial correlation to the downregulation of the pro-inflammatory markers. On the contrary, white onion peel along with root samples of the four cultivars demonstrated negative PC1 values away from pro-inflammatory markers. Red onion peel demonstrated a strong spatial correlation with inhibiting IL-1*β*. Regarding IL-6, the highest spatial correlation was observed in case of golden yellow onion peel. Meanwhile, copper-yellow onion peel showed the highest spatial correlation with inhibiting IFN-*γ* and TNF-*α*.

In order to untangle the main anti-inflammatory bioactive metabolites that are likely to drive the discrimination of the tested onion samples, correlation analysis was attempted. As shown in Fig. 5B, *γ*-glutamyl glutamine, *γ*-glutamyl-methionine sulfoxide, quercetin 3-*O*-glucoside, peondin 3-*O*-glucoside isorhamnetin 3,4’-*O*-diglucoside sativanone, 3-methoxytyramine, pipecolic, succinic, stearic acids were the main bioactive metabolites positively correlated to the suppression of IL-1*β* being mostly abundant in red onion peel. On the other hand, *γ*-glutamyl-*S-*(prop-2-enyl) cysteine sulfoxide, *γ*-glutamyl-*S-*(propyl) cysteine sulfoxide, vanillic acid, rosmarinic acid, taxifolin, 3’-methoxylunularic acid, 3’-hydroxymelanettin, quercetin dimer, quercetin dimer adduct with methyl protocatechuate and oleic acid were positively correlated with decreased levels of IL-6, and these compounds were detected in substantial amounts in peel samples of golden yellow onion (Fig. 5B). Regarding IFN-*γ* and TNF-*α*, (iso)alliin, glycolic acid, *p*-coumaroyl glycolic acid, ascorbic acid, 3’-methoxylunularic acid, quercetin 4’-*O*-glucoside, isorhamnetin 4’-*O*-glucoside, alliospiroside D, were the metabolites positively correlated to the downregulation of these pro-inflammatory markers (Fig. 5B). These metabolites exhibited the highest abundance in copper-yellow onion peel.

## Discussion

### Chemical profiling using UPLC–ESI–MS/MS

UPLC–MS/MS analysis of peel and root extracts of the tested onion cultivars revealed **103** metabolites of diverse chemical classes, e.g., amino acids, peptides, phenolic acids, flavonoids, saponins, and fatty acids. The identification of compounds was accomplished by comparing their retention time (t_R_), quasi-molecular ions and their MS/MS fragment ions with external standards whenever possible (Table 1). Additionally, data previously reported in literature and databases such as MassBank and Dictionary of Natural Products were also utilized to achieve metabolite annotation with a high level of confidence (level II) [17]. The different classes of annotated compounds are listed below, along with a discussion of a few examples from each class. The structures of the discussed compounds are provided in (Figure S2) in supplementary material.

#### Amino acids and peptides

This class of compounds was represented by peaks **1–8, 10, 14, 18–21, 24, 26, 27, 30, 33, 35, 39, 44, 46** and **69**. Compound **6** showed a quasi-molecular ion peak at 293 Da [M − H] ^−^. The daughter ion detected at 128 Da indicated glutamine loss along the amide linkage. Other daughter ions were observed at 275 Da [M − H − 18] ^−^ representing the loss of H_2_O, and at 164 Da attributed to deprotonated methionine sulfoxide after loss of (C_5_H_8_NO_3_) moiety. Hence, compound **6** was identified as γ-glutamyl-methionine sulfoxide [26]. Compound **8** showed a quasi-molecular ion peak [M + H] ^+^ at 178 Da along with two daughter ions at 137 Da and 120 Da due to the elimination of C_3_H_5_ and C_3_H_6_O moieties, respectively. Another daughter ion was detected at 161 Da indicating the loss of NH_3_. Based on the above mentioned data, compound **8** was annotated as (iso)alliin [27].

#### Flavonoids and anthocyanins

UPLC-MS analysis of peel and root extracts of the four onion cultivars enabled the identification of **34** flavonoids belonging to different classes. Among these, flavonols constituted the most predominant class (compounds **51, 52, 53, 56, 58, 59, 61, 62, 63, 64, 71, 72, 73, 75, 79, 81, 83, 86, 88 & 89**) coinciding with literature [1, 10]. Additionally, anthocyanins (**28, 47, 48 & 50**), flavones (**43**), flavanols (**45**), dihydroflavonols (**55 & 76**), flavanones (**66, 80 & 93**), isoflavanones (**87 & 90**), neoflavones (**77**) were also characterized. Peak **51** gave a quasi-molecular ion peak at 625 Da [M − H] ^*–*^ along with daughter ions at 463 Da [M *–* H – 162] ^–^ and 301 Da [M – H – (2 × 162)] ^−^ corresponding to the subsequent loss of one and two molecules of glucose, respectively. Other fragment ions were detected at 151 Da, and 179 Da attributed to retro Diels-Alder (rDA) cleavage of ring C of the aglycone moiety (301 Da) [28, 29]. Hence, the compound was identified as quercetin diglucoside. Regarding identified anthocyanins, three were acylated derivatives of cyanidin monoglucoside (**28, 47& 48**) and one was peonidin derivative (**50**). Compound **48** showed precursor ions at 533 Da [M − 2 H] ^−^ & 551 Da [M − 2 H + H_2_O] ^−^. Fragment ions were observed at 447 Da [M − 2 H − malonyl] ^−^ and at 285 Da ascribed to cyanidin aglycone [Aglycone − 2 H] ^−^. Another fragment ion was observed at 241 Da resulting from the cleavage of aglycone. Hence, compound **48** was annotated as cyanidin 3-*O*-malonylglucoside in line with previously reported data [30]. Compound **53** displayed a very intense quasi-molecular ion peak [M − H] ^−^ at 317 Da. It showed a dehydroxylated species at 299 Da [M − 17] ^–^ along with other daughter ions at 191 Da, 207 Da and 273 Da which established the identity of compound as 2-(3,4-dihydroxybenzoyl)-2,4,6- trihydroxy-3(2 H)-benzofuranone [31]. Both compounds **64 & 79** presented pseudo-molecular ions at 763 Da and were readily assigned as hexoside derivatives of quercetin dimer. Both compounds showed a daughter ion at 601 Da that resulted from the loss of a hexoside residue. Another fragment ion was detected at 611 Da generated by rDA cleavage along with a characteristic ion at 299 Da assigned to quercetin *O*-diquinone ion formed upon rDA scission of the dioxane ring [31, 32].

#### Phenolic and organic acids

A total of 17 phenolic and organic acids were characterized; seven hydroxycinnamic acid derivatives were identified in peaks **12, 13, 15, 17, 32, 36 & 37**, three hydroxybenzoic acid derivatives were annotated in peaks **23, 17 & 41**, whereas organic acids were represented by compounds **9, 11, 16, 22, 29, 31 & 34**.

Compound **22** showed a quasi-molecular ion peak [M − H] ^–^ at 191 Da along with its diagnostic ions at 85 Da and 111 Da corresponding to [M − H − (2 x CO_2_ + H_2_O)] ^−^ and [M − H − (2 x H_2_O + CO_2_)] ^−^, respectively. This compound was annotated as citric acid [33]. Compound **23** exhibited its quasi-molecular ion peak at 167 Da along with its characteristic daughter ions at 153 Da [M − H − 14] ^−^, 137 Da [M − H − 30] ^−^ and 123 Da [M − H − 44] ^−^, corresponding to the loss of CH_3_, OCH_3_ and CO_2_ from the precursor ion, respectively. The compound was assigned as vanillic acid [34].

#### Saponins

The *Allium* genus is well known for the abundance of steroidal saponins which were reported by various studies to be responsible for many of the health beneficial activities exerted by the members of this genus. Onion steroidal saponins are mostly spirostanol or furostanol derivatives [35]. Several spirostanol saponins were identified in peaks **82, 91, 92, 94, 99 & 103**. On the other hand, furastanol saponins with different oxygenation patterns were assigned in peaks **49, 54, 57, 60, 68 & 74**, which eluted in earlier portion of the chromatogram. In addition, one pregnane type saponin was characterized in compound **78**. Also, one triterpenoid saponin of ursane skeleton was assigned in peak **85**. Compound **49** produced a quasi-molecular ion peak [M – H] ^−^ at 725 Da. The precursor ion generated fragments at 593 Da, 447 Da and 429 Da by the consecutive loss of pentosyl, rhamnosyl and water moieties. On the basis of these features, compound **49** was annotated as tropeoside B [36].

#### **Hydroxycinnamic acid amides (**HCAAs)

This class of compounds is formed by conjugation of amines with hydroxycinnamic acids and is represented by three peaks: **65, 67 & 70**. The detected compounds generated product ions either at 177 Da or 147 Da ascribed to protonated feruloyl or coumaroyl moieties, respectively. Other fragment ions were observed at 138 Da and 121 Da which corresponded to protonated tyramine moiety and vinylphenol ion formed upon the loss of NH_3_ from the former. For instance, compound **65** exhibited a quasi-molecular ion peak [M + H] ^+^ at 284 Da. It gave rise to fragment ions at 147 Da, 138 Da and 121 Da and accordingly, it was characterized as *N*-*p*-coumaroyltyramine [37].

#### Fatty acids

Fatty acids eluted mostly in the late elution part of the chromatogram (t_R_ 21–25 min); saturated fatty acids were identified in peaks **96, 97 & 98**, whereas unsaturated fatty acids were represented by compounds **95, 100, 101 & 102**. Compound **95** was detected at 295 Da representing its quasi-molecular ion peak [M − H] ^−^. Fragmentation of the precursor ion generated daughter ions at 277 Da, 251 Da and 239 Da indicating the loss of H_2_O, CO_2_ and C_4_H_8_O_4_, respectively. In detail, compound **95** was characterized as oxo-octadecenoic acid [38].

### Multivariate data analysis

To analyze UPLC-MS/MS data in a more holistic way, HCA was implemented to investigate the relative metabolites variation among the different onion samples. Moreover, PCA score scatter and loading plots were also implemented to define the clustering of the tested samples and the compounds mediating such gathering [39]. The HCA heatmap and score scatter plot revealed notable disparities among the tested samples. As depicted in Figs. 1 and 2, with the exception of white onion, the samples from the same organ clustered closely. In contrast, root and peel samples from the same cultivar remained separate from each other. This suggests the effect of the organ rather than the cultivar on the chemical profile of tested samples. By referring to Fig. 1, the metabolites that were commonly detected in peel extracts of the three colored cultivars were flavonoids including flavonols, cyanidins and quercetin oxidized derivatives such as quercetin, kaempferol, kaempferol *O*-rhamnosyl-hexoside, isorhamnetin, isorhamnetin 3,4’-*O*-diglucoside, cyanidin 3-*O*-acetylglucoside, quercetin dimer hexoside isomer, 2-(3,4-dihydroxybenzoyl)-2,4,6-trihydroxy-3(2 H)-benzofuranone and quercetin trimer. This could explain the proximate grouping of these samples together (group A). Moreover, the close placement of root samples of the three colored cultivars could be attributed to the common distribution of alliospiroside A, alliospiroside B, ceparocide I, ascalonicoside A, palmitic acid, and linolenic acid in their extracts (subgroup B1). As for peel and root samples of white onion, the less enrichment of their extracts with metabolites compared to other samples may be the reason behind their clustering together (subgroup B2). Further, some metabolites were common in these two samples such as sinapic, tartaric, pyroglutamic acids and ornithine.

By referring to Fig. 2, the clustering pattern of root samples of the four cultivars was more consistent than that of peel samples indicating a higher similarity in their chemical profiles. Moreover, chemical profiles of peel extracts of the yellow onion cultivars were closely related as they clustered together. On the other hand, the chemical profile of white onion peel showed greater variation when compared to peel samples of the other tested cultivars since it clustered very close to root samples suggesting a relative similarity of its chemical profile to those of root samples. These results indicated a more pronounced effect of organ rather than cultivar on the segregation of tested samples.

### Evaluation of the PDE-5 inhibitory and anti-inflammatory activities

In ED, upregulation of PDE-5 catalyzes the breakdown of the cGMP and also decreases NO levels in the endothelial cells, thereby hindering vasodilation of blood vessels, which eventually impairs penile erection [40]. Chronic, mild inflammation plays a significant role in the development of ED since it can damage the inner lining of blood vessels, leading to a condition called endothelial dysfunction [12, 13]. This condition can specifically affect the blood vessels and nerves that are essential for erectile function. The erectogenic properties of peel and root extracts of the four onion cultivars were examined via evaluating their inhibitory potentials to PDE-5 enzyme relevant to ED. All the tested onion peel and root samples exerted promising inhibition to PDE-5 enzyme showing varying potencies. The highest activity was displayed by golden yellow root, which showed efficacy comparable to that of sildenafil, closely followed by red onion root. Regarding peel samples, peel extracts of copper-yellow and red onions showed the highest efficacies with very close IC_50_ values (Table 2). The obtained findings agreed with the study carried out by Lines & Ono, 2006 which identified FRS 1000, a beverage prepared from onion peel having quercetin as the main ingredient, as an inhibitor of PDE-5 [41]. Moreover, onion peel extract enhanced the motility and viability of spermatozoa and exerted a beneficial effect on male fertility via altering voltage-gated proton potentiation in the human embryonic kidney cell line HEK293 [42]. In a study by Allouh et al., fresh onion juice significantly increased testosterone levels in the blood and enhanced sexual behavior in male rats with normal sexual potency as well as those suffering from paroxetine-induced sexual dysfunction [43]. Further, onion extract effectively mitigated the negative impacts of cadmium on testicular oxidative damage and spermiotoxicity in rats, possibly due to its potential in decreasing lipid peroxidation and enhancing the antioxidant defense system [44]. In another study by Khaki et al., fresh onion juice notably enhanced sperm motility, viability, and concentration, and also significantly boosted the levels of serum testosterone in Wistar male rats [45]. It is worth mentioning that this study is the first one evaluating onion roots for this activity.

For the evaluation of the anti-inflammatory activity of onion samples, MTT test was utilized to evaluate safety of the tested extracts where CC_50_ values were determined for the tested samples as well as the standard piroxicam. Inflammation was induced in normal WBCs using LPS which is a protein obtained from the outer membrane of Gram-negative bacteria. Meanwhile, it is a toxic agent that elicits inflammation via stimulating the production of cytokines by WBCs, including TNF-*α*, IL-1*β*, IL-6, IL-12 and IFN-*γ* [46]. All the tested onion peel and root samples demonstrated a superior safety profile in comparison to piroxicam, with the root samples exhibiting higher safety profiles compared to the peel samples (Table 2). Moreover, all the tested samples demonstrated promising anti-inflammatory potentials, exhibiting variable efficacies (Table 2), where most of them were more effective than piroxicam. Additionally, all the tested samples significantly decreased the upregulation of the four proinflammatory markers in LPS-treated WBCs (Fig. 3). Interestingly, the levels of the pro-inflammatory cytokines were reduced to a greater extent compared to the levels exhibited by piroxicam. The samples exhibited varying efficacies against the different pro-inflammatory markers, highlighting their diverse effects on the inflammatory response. Peel extract of copper-yellow onion exhibited the highest potency in suppressing the TNF-*α* and IFN-*γ* genes. On the other hand, peel extracts of red and golden yellow onions were found to be the most effective in downregulating the IL-1*β* and IL-6 genes (Fig. 3**)**. It is worth mentioning that peel samples exhibited superior efficacy compared to root samples, which designates the significant impact of organ type on the anti-inflammatory activity displayed by the tested samples.

The findings of this study are corroborated by a previous report in which onion peel extract effectively downregulated the LPS-induced overexpression of TNF-*α* in a HT-29 cell model [47]. In another study by Ahn et al., the hot water extract of onion peel showed a dose-dependent decrease in NO levels and inhibited IL-6, TNF-*α*, and IL-1*β* pro-inflammatory cytokines in the murine macrophage cell line RAW 264.7 in vitro and suppressed croton oil-induced ear swelling in ICR mice in vivo [48]. Additionally, onion peel extract exerted significant anti-inflammatory and cytotoxic activities on LPS-stimulated human colon carcinoma cells [47]. Moreover, this work is concordant with a previous study claiming that red onion scales extract exerted potent anti-inflammatory and immunomodulatory potentials and significantly ameliorated the overexpression of IL-6, IL-8 and TNF-𝛼 in Atypical prostatic hyperplasia-induced rats [49]. Additionally, the ethanolic extract of onion roots was reported to display a significant anti-inflammatory activity in carrageenan-induced paw edema model in rats [50].

It was revealed that among the peel samples, copper-yellow onion showed the lowest IC_50_ value for PDE-5 inhibitory activity and also the lowest EAIC value for anti-inflammatory activity, suggesting it might be the most potent in relieving ED and associated inflammation. Regarding root samples, golden yellow onion exhibited the lowest IC_50_ value for PDE-5 inhibitory activity, together with a low EAIC value for anti-inflammatory activity indicating its effectiveness in both aspects. Consequently, it could be concluded that there is a strong correlation between the PDE-5 inhibitory and anti-inflammatory activities exerted by the tested onion samples.

### Determination of the PDE-5 inhibitory activity and anti-inflammatory biomarkers using PLSR analysis

#### Determination of the PDE-5 inhibitory activity biomarkers

To examine the subsequent grouping of the tested extracts in relation to their PDE-5 inhibitory activity, as well as to determine the biomarkers mediating such activity, a PLS model was constructed. The created model showed accepted degrees of stability and reliability. The biplot (Fig. 4A) showed a strong spatial correlation between PDE-5 inhibitory activity and root samples of golden yellow and red onions as well as peel samples of red and copper yellow onions, since they were the most active extracts. The coefficient plot (Fig. 4B) revealed six compounds positively correlated to the PDE-5 inhibitory activity. These compounds exhibited high abundance in the active samples as follows: cyanidin 3-*O*-(malonyl-acetyl)-glucoside and quercetin adduct with methyl protocatechuate recorded considerable amounts in root extracts of red and golden yellow onions. Meanwhile, methionine, morin and quercetin dimer hexoside were detected in a high concentration in peel and root extracts of red onion, while diosgenin accumulated in high amounts in root extract of golden yellow onion (Fig. 1). This could establish the paramount relevance of these bioactive metabolites to the substantial PDE-5 inhibitory potential of such extracts that ultimately distinguished them from the less active samples.

The identified biomarkers were previously shown to inhibit PDE-5 enzyme. Anthocyanins were demonstrated to possess PDE-5 inhibitory potential and served as good therapeutic candidates in premature ejaculation [51]. Moreover, amino acids exerted inhibitory activity against enzymes relevant to ED including PDE-5 [52]. Quercetin and other flavonoids showed remarkable in vitro PDE-5 inhibitory activities [40]. Further, diosgenin was shown to restore the normal levels of cGMP in doxorubucin-treated mice via modulation of PDE-5 activity [53]. Also, it substantially improved serum testosterone level and enhanced sperm count, viability, and motility in diabetic rats [54].

#### Determination of the anti-inflammatory biomarkers

A PLS model was constructed to investigate the grouping of onion samples in terms of their anti-inflammatory potential as well as define the biomarkers positively correlated to such activity. The obtained findings revealed the good predictive ability and goodness of fit of the constructed model. The biplot (Fig. 5A) showed a strong spatial correlation between the downregulation of the pro-inflammatory markers and peel samples of the colored cultivars. Red onion peel demonstrated a strong spatial correlation with inhibiting IL-1*β*. On the other hand, golden yellow onion peel showed the highest spatial correlation to IL-6 inhibition. Meanwhile, copper-yellow onion peel displayed the highest spatial correlation with IFN-*γ* and TNF-*α* suppression. These results suggest a substantial difference in the effect of peel extracts of the tested cultivars on the inhibition of the pro-inflammatory markers. It was clearly evident that peel samples of the tested cultivars were significantly more active in suppressing the measured pro-inflammatory markers than root samples with the exception of white onion peel, since it exerted lower anti-inflammatory potential when compared to peel samples of the colored cultivars. This could be attributed to the less enrichment of its extract with bioactive metabolites compared to peel extracts of the colored cultivars as revealed from its base peak chromatogram (Figure S1). The findings notably demonstrated that the organ significantly influenced the anti-inflammatory activity of the tested samples, surpassing the impact of the cultivar. This aligns with the earlier findings that the organ had a more pronounced effect on the chemical composition of the samples compared to the cultivar (Sect. 4.2). The coefficient plot (Fig. 5B) unveiled the main bioactive metabolites positively correlated to proinflammatory markers inhibition. These compounds displayed high abundance in peel samples of the three colored cultivars. These bioactive metabolites belonged to diverse chemical classes such as amino acids (e.g., methionine and pipecolic acid), sulfur and non-sulfur containing γ-glutamyl peptides (e.g., *γ*-glutamyl glutamine and *γ*-glutamyl-*S*-(propyl) cysteine sulfoxide), flavonoids and anthocyanins (e.g., quercetin 4’-*O*-glucoside, isorhamnetin 3,4’-*O*-diglucoside, sativanone, taxifolin, and 3’-hydroxymelanettin, peondin 3-*O*-glucoside), phenolic and organic acids (e.g., vanillic, rosmarinic, ascorbic, succinic, and glycolic acids), fatty acids (e.g., stearic and oleic acids), and saponins (e.g., alliospiroside D).

Upon searching the literature, the identified bioactive metabolites were previously shown to possess anti-inflammatory potential. γ-Glutamyl di-peptides and organo-sulfur compounds have been previously reported to exhibit potent anti-inflammatory activities and reduce the expression of pro-inflammatory markers [55–57]. Moreover, vanillic, ascorbic, succinic, rosmarinic, glycolic and stearic acids have been identified to act as effective anti-inflammatory agents [52, 58–63]. Additionally, anthocyanins, glycosides of quercetin and isorhamnetin displayed marked anti-inflammatory activities in various in vivo and in vitro models and were effectively able to suppress the production of various pro-inflammatory mediators [64–69]. Further, sativanone, taxifolin and 3’-hydroxymelanettin were reported by various studies to possess anti-inflammatory potential [70–74]. Steroidal saponin glycosides were also shown by various studies to exert pronounced anti-inflammatory activity via inhibiting the production of pro-inflammatory cytokines [75, 76].

## Conclusion

The surge in onion consumption and the growth of the processed onion market have led to a substantial increase in onion waste, making it indispensable to find ways for its valorization. In this study, UPLC-MS/MS analysis combined with chemometric tools enabled efficient metabolic profiling of peels and roots of four onion cultivars (red, copper-yellow, golden yellow and white onions). A significant variation in the chemical composition was observed between different samples, and it was revealed that organ showed more pronounced effect on the chemical profiles of samples compared to cultivar. All the tested extracts exhibited promising PDE-5 inhibitory and anti-inflammatory activities showing varying efficacies. The organ type significantly influenced the biological activity of the samples, highlighting the importance of choosing the right organ to maximize the desired efficacy. Cyanidin 3-*O*-(malonyl-acetyl)-glucoside and quercetin dimer hexoside showed the strongest correlation with PDE-5 inhibition. Meanwhile, γ-glutamyl-methionine sulfoxide, taxifolin, quercetin 4’-*O*-glucoside and isorhamnetin 4’-*O*-glucoside were the main metabolites positively correlated to the inhibition of IL-1*β*, IL-6, IFN-*γ* and TNF-*α* pro-inflammatory markers, respectively. This study shed the light on onion waste as rich source of bioactive constituents with potential uses in erectile dysfunction and inflammation. These unique bioactive compounds call for further in vitro and in vivo studies and could be the keystone of an evidence based investigation to discover new effective anti-PDE-5 and anti-inflammatory drug candidates.

### Electronic supplementary material

Below is the link to the electronic supplementary material.

Supplementary Material 1

## Data Availability

The datasets used to support this study are available from the corresponding author upon request and after satisfying ethical requirements for their release.

## Abbreviations

ANOVA: Analysis of variance
cGMP: Cyclic guanosine monophosphate
CID: Collision induced dissociation
DMSO: Dimethyl sulfoxide
EAICs: Effective anti-inflammatory concentrations
ED: Erectile dysfunction
ESI: Electrospray ionization
HCA: Hierarchical cluster analysis
HCAAs: Hydroxycinnamic acid amides
IFN-γ: Interferon-γ
IL-1β: Interleukin-1β
IL-6: Interleukin-6
LPS: Lipopolysaccharide
MTT: 3-(4, 5-dimethylthiazol-2-yl)-2, 5-diphenyl tetrazolium bromide
NO: Nitric oxide
PCA: Principal component analysis
PCR: Polymerase chain reaction
PDE-5: Phosphodiesterase-5
PLSR: Partial least squares regression
Q^2^: Cross validation redundancy value
QqQ-MS/MS: Triple quadruple-Tandem mass spectrometry
R^2^: Coefficient of determination
rDA: Retro Diels-alder
SD: Standard deviation
TNF-α: Tumor necrosis factor-α
t^R^: Retention time
UPLC-MS: Ultra-performance liquid chromatography-mass spectrometry
WBC’s: White blood cells
