# Supplementary material for: Insights into bioactive constituents of onion (Allium cepa L.) waste: a comparative metabolomics study enhanced by chemometric tools
Source: BMC Complement Med Ther. 2024 Jul 15;24:271. doi: 10.1186/s12906-024-04559-2 (PMC11250982; doi:10.1186/s12906-024-04559-2)
Supplement: Supplementary file 1 — Supplementary Material 1 [file 12906_2024_4559_MOESM1_ESM.docx]

**2.1. Reagents and apparatus**

Phosphodiesterase 5 (PDE-5) enzyme (Recombinant, bovine) was purchased from Merck Millipore. The reference standards alliin, ferulic acid, quercetin, *β*-chlorogenin and palmitic acid, the external standards used for the annotation of the compounds, HPLC grade acetonitrile, formic acid, tris-HCl buffer, *p*-nitrophenyl phenylphosphate, sildenafil, dimethyl sulfoxide (DMSO), SYBR green master mix, trypan blue, Lipopolysaccharides (LPSs), and MTT (3-(4,5-dimethylthiazol-2-yl)-2,5-diphenyl tetrazolium bromide) dyes, ammonium chloride lysing solution, RNA and cDNA extraction kits, reverse transcriptase, nuclease free water and RNase inhibitor were purchased from Sigma Aldrich (St. Louis, MO, USA). Piroxicam was purchased from Merck (Darmstadt, Germany). L-glutamine and Fetal Bovine serum, Roswell Park Memorial Institute (RPMI) 1640 Medium, were obtained from Lonza (Belgium). dNTPs (deoxynucleotide triphosphate) and dT primer, were procured from Thermo Fisher Scientific. Freeze dryer (Alpha 1–2 LD, Christ, Germany), rotary evaporator (Rotavap Buchi 461) and CFX96™ Real-Time System (BIO-RAD, USA) were utilized. The UPLC system encompassed a Waters Acquity QSM pump, a LC-2040 (Waters Corporation) autosampler, degasser and Waters Acquity CM detector. Samples were separated using a Waters Acquity UPLC BEH C18 column (1.7 µm particle size – 2.1 × 50 mm). Waters Corporation, Milford, MA01757 U.S.A, mass spectrometer was utilized. ESI-MS positive and negative ion acquisition mode was carried out on a XEVO TQD triple quadruple instrument.

**2.3.4. Semi-quantitation of metabolites using standard solutions**

In order to investigate the variability in the chemical profiles of peel and root extracts of the tested onion cultivars and to unravel the organ effect on the quality and quantity of the identified metabolites, all the detected metabolites were analyzed in each tested extract based on mean peak area computation using the calibration curves of corresponding standards and the results were readily presented as (mg standard equivalents/g dry extract) (**Table S4**). The identified compounds were subjected to semi-quantitative analysis based on their chemical class, using standard compound solutions (1 mg/10 mL) evaluated under the same MS-conditions as previously indicated. Representatives of these chemical classes were alliin, ferulic acid, quercetin, *β*-chlorogenin and palmitic acid (Sigma-Aldrich (St. Louis, MO, USA). Each of the standard solutions was prepared by dissolving an accurate weight of the standard (10 mg) in 10-mL HPLC-grade methanol. After that, the solution was serially diluted to the working concentrations over the reliable range 0.0125 – 0.75 mg/mL. 5 μL from each concentration level were injected into the chromatographic system in duplicates for establishment of calibration curves. Validation parameters like linearity, limit of detection (LOD) and limit of quantiﬁcation (LOQ) were assessed based on FDA guidelines on bioanalytical method validation (**Table S3**) [1].

**2.4. PDE-5 inhibition assay**

The potential of the extracts to inhibit PDE-5 enzyme activity *in vitro* was examined spectrophotometrically by following the change in absorption at 400 nm using *p*-nitrophenyl phenylphosphate as substrate according to the procedure developed by Kelly & Butler, 1977 [2] with slight modification by Oboh et al., 2017 [3]. In brief, the reaction mixture was prepared by adding 5 mM of the substrate (*p*-nitrophenyl phenylphosphate), 100 μL of enzyme, 20 mM tris-HCl buffer (pH 8.0), and the test inhibitor solution. After incubation at 37 °C for 10 min, the intensity of *p*-nitrophenol formed was measured as change in absorbance at 400 nm. The control experiment was carried out without the test sample. Sildenafil (25 µg/mL): a well-known PDE-5 inhibitor, was set up as a positive control for the inhibition test. All determinations were performed in triplicate thus inhibition percentage was expressed as the mean of three observations. PDE-5 inhibitory activity was expressed as percentage inhibition of the enzyme in the above assay mixture system, calculated by the formula below:

[ (Abs **_control_** – Abs **_sample_)** / Abs **_control_**] x 100 where Abs **_control_** is the absorbance of the control solution without the tested samples, Abs **_sample_** is the absorbance of the tested samples solution.

**2.5. Evaluation of cytotoxicity and anti-inflammatory activity of peel and roots extracts of the tested onion cultivars**

**2.5.1. Human white blood cells isolation and cultivation**

Based on Mosmann 1983 method, a whole blood specimen was obtained and transferred to a sterile heparin tube, then 1mL blood was taken into a centrifuge tube (15 mL) [4]. Thereafter, fresh cold ammonium chloride lysing solution was added to the tube capacity and it was inverted for ~10 minutes at room temperature until the liquid became clear red. Centrifugation of samples was carried out at temperature of 4 ^o^C for 10 min at 2000 rpm followed by decantation of the supernatant and the tubes were allowed to drain, suspension of the pellets (WBCs) was done in 10 mL cold phosphate buffer saline (137 mM NaCl, 2.7 mM KCL, 10 mM Na_2_HPO_4_ and 10 mM KH_2_PO_4_) pH 7.4, recentrifuged and pellets were resuspended in RPMI culture medium containing 10% fetal bovine and 2% L-glutamine. The dye exclusion method was used for the assessment and counting of WBCs [5]. Fifty µL of the cell suspension was mixed with an equal volume of 0.5% trypan blue staining solution followed by their loading onto hemocytometer. Counting was done for both viable unstained and nonviable stained cells.

**Calculation**

**N / mL = mean of WBCs counting x 10^4^ x D**

N: Number of viable or nonviable cells

D: Sample dilution (1:1 with the trypan blue).

**% of cell viability = Number of viable cells x 100**

**Total number of cells**

In order to use the cells for assays, at least 90% of the cells must be viable, after cultures were incubated in CO_2_ incubator for six days. WBCs were seeded as 100,000 cells/ well (96 well cell culture plate) and incubated in a CO_2_ incubator (37 °C, 5% CO_2_, and 90% relative humidity).

**2.5.2. Evaluation of cytotoxicity of the tested extracts compared to the standard anti-inflammatory drug (piroxicam) (MTT assay)**

According to the MTT (3-(4, 5-dimethylthiazol-2-yl)-2, 5-diphenyl tetrazolium bromide) assay, metabolically active living cells were detected by mitochondrial succinate dehydrogenase that converts the MTT into a dark purple insoluble formazan. Solubilization of cells was done using DMSO and the quantitation of the released, solubilized formazan was done spectrophotometrically aiming at checking the cytotoxicity of the test sample and piroxicam against human WBCs [4]. Different concentrations (0, 3.125, 6.25, 12.5, 25 and 50 μg/mL) of the studied samples were plated in 96-well cell culture plate with 200 µL of cultured medium that contained 100,000 WBCs/well in RPMI medium without fetal bovine serum or piroxicam (standard anti-inflammatory drug). After that, the plate was incubated for 72 h in a CO_2_ incubator (37 °C, 5% CO_2_, and 90% relative humidity). After the incubation, 20 μL of MTT solution was added to each well and then plates were incubated for 3 h in a CO_2_ incubator to allow the MTT to react. After incubation, centrifugation of the additional plates was done at 1650 rpm for 10 min and the medium was discarded. The formazan crystals (MTT byproduct) were re-suspended in 100 μL DMSO and reading was measured at a wavelength of 570 nm in order to detect cell cytotoxicity 50 (CC_50_) value that results in 50% cell death**.**

**The % viability was calculated as follow: (A_T_** – **A_b_ / A_C_** – **A_b_) x 100**

**A_T_** = mean absorbance of cells treated with different concentrations of extract.

**A_C_** = mean absorbance of control untreated cells with culture medium only

**A_b_** = mean absorbance of cells treated with vehicle of the sample (RPMI without fetal bovine serum)

Cell cytotoxicity 50 (CC_50_) is the concentration of the drug or the extract that results in 50% cell death, which was calculated by the GraphPad software (GraphPad Software Inc, California) using the % viability calculated from the serial dilutions of the test sample.

**2.5.3. Detection of the effective anti-inflammatory concentrations (EAICs) of the used treatments in Lipopolysaccharides (LPSs)-stimulated human WBC's culture**

In the present experiment, LPS acts as a common inflammatory inducer and causes abnormal up-regulation in the proliferation of human leukocytes. The abnormal increase in cell proliferation can be used as a marker of inflammation. This assay was performed according to MTT test (please refer to section 2.5.2).

**Procedure**

In a 96 well plat, A volume of 50 μL of the culture medium that contained 100,000 of human WBCs was dispensed per well. The inflammation was induced by adding 50 μL of LPS to the plated cells and incubated in CO_2_ incubator. After 24 h, the plate was centrifuged at 1650 rpm for 5 min and the supernatants were discarded and then 200 µL of serial concentrations (0, 3.125, 6.25, 12.5, 25 and 50 μg/mL in culture media) of the crude extracts or the standard anti-inflammatory drugs piroxicam were added. The control cells contained cell culture medium only. The plates were incubated for an additional 72 h in CO_2_ incubator. After 72 h of incubation, the cell proliferations were measured using MTT (as previously illustrated in section 4.2). Stimulation index (SI) was used to assess the cell proliferations.

**Stimulation index** = (mean absorbance of LPS-stimulated cells or LPS-stimulated cells treated with different concentrations of natural product/ mean absorbance of control untreated cells).

The effective anti-inflammatory concentration (EAICs) of test samples can be defined as the concentration that is able to bring back the abnormal proliferation of LPS-stimulated cells to normal proliferation of control untreated cells (SI = 1) and were calculated using the GraphPad software.

**2.5.4. Extraction of RNA of untreated and treated LPS-stimulated human WBCs and cDNA synthesis**

In 50 µL of solution R1, cell pellets were suspended and mixed for 30 s, then their incubation was done at room temperature for 1 min. 300 µL of solution R2 were added and mixed for 30 s then the centrifugation was done at 4ºC for 3-5 min. Into a spin column, the supernatant was transferred and centrifuged for 30 s at 14000 rpm at 4ºC. 300 µL of working wash buffer were added into the spin column after discarding the flow-through and, centrifuged for 30 s and this step was repeated in twice. Centrifugation of the spin column was done for 1min at 10,000 rpm then it was transferred to a sterile 1.5 mL micro centrifuge tube. To the centre of the membrane, 30 µL of elution buffer were added and incubated at room temperature for 1 min followed by their centrifugation for 30 s at 14000 rpm at 4ºC. Finally, determination of the optical density (OD) of the extracted RNA was done through measuring the absorbance and purity at A260 and A260/A280 nm, respectively using spectrophotometer and kept in -80°C until real time PCR.

Two µg of total RNA or nuclease-free water and 1 µL of oligo dT primer were added to nuclease-free water in a total volume of 12 µL in PCR tubes, then they mixed gently. Centrifugation, incubation at 65ºC for 5 min in PCR machine was carried out, then they were placed back on ice immediately. The gentle mixing of 4 µL of 5X reaction buffer, 1 µL of RNase inhibitor, 2 µL of dNTPs mix and 1 µL of reverse transcriptase or 1 µL of nuclease-free water instead of reverse transcriptase for reverse transcriptase negative control with previous mixture was performed. After that, spin down and incubation for 60 min at 42ºC followed by heat inactivation at 70ºC for 5 min in PCR machine was carried out.

**2.5.5. Determination of IL-1*β*, IL 6, TNF and INF-*γ* expression level by real time polymerase chain reaction (PCR)**

13 µL of 2 x SYBR green master mix was mixed with 5 µL of cDNA, 0.5 µL of 10 pmoles/mL forward primer and 0.5 µL of pmoles/mL reverse primer for each primer in PCR tubes. As for the reference tube, 0.5 µL of 10 pmoles/mL forward primer of *β*-actin and 0.5 µL of 10 pmoles/mL for reverse primer of *β*- actin were added. In order to assess for reagent contamination or primer dimers, another tube was used as a non-template control (NTC) by adding 1 µL of nuclease-free water instead of template used. After that, the gentle mixing of the tubes with 6.5 µL nuclease free water without creating bubbles was done and then they were spined for few seconds. In the cycler, samples were placed, and the program was started as follows; initial denaturation (1 cycle of 95ºC for 10 min), followed by denaturation (40 cycles of 95ºC for 15 sec), annealing (at 60ºC for 30s) and extension (at 72ºC for 30s). The effect of LPS and extracts on gene expression was expressed as fold change in gene expression which was calculated according to the following equations:

**Expressions fold levels of gene calculated by**

ΔCt _normal_ = Ct _normal untreated cells_ – Ct _reference_

ΔCt _tested plant extract_ = Ct _tested plant extract-treated cells_ – Ct _reference_

ΔCt _induced_ = Ct _LPS-exposed cells_ – Ct _reference_

**In case of genes:**

ΔΔCT _tested plant extract_ = ΔCt _tested plant extract_ – ΔCt _normal_

ΔΔCT _induced_ = ΔCt _induced_ – ΔCt _normal_

**In case of GAPDH:**

ΔΔCT _tested plant extract_ = ΔCt _normal_ – ΔCt _tested plant extract_

ΔΔCT _induced_ = ΔCt _normal_ – ΔCt _induced_

**Fold change in gene expression = log (2^-ΔΔCT^)**

**Where: Ct _tested plant extract_**: threshold cycle value of genes of extracted mRNA of plant extract treated-LPS-stimulated WBCs which is defined as the cycle number at which the fluorescence generated within a reaction crosses the fluorescence threshold.

**Ct reference**: threshold cycle value of GAPDH which is used for normalization.

**Ct _normal_**: threshold cycle value of genes of extracted mRNA of untreated control WBCs

**Ct _induced_**: threshold cycle value of gene of extracted mRNA of LPS-stimulated WBCs

The primers used:

| TNF-*α* | F-CTCTTCTGCCTGCTGCACTTTG |
| --- | --- |
|  | R- ATGGGCTACAGGCTTGTCACTC |
| IL-6 | F, 5′-TGAACTCCTTCTCCACAAGCG-3′ |
|  | R, 5′-TCTGAAGAGGTGAGTGGCTGTC-3′ |
| IL-1*β*, | F, CCACAGACCTTCCAGGAGAATG |
|  | R, GTGCAGTTCAGTGATCGTACAGG |
| INF-γ | F, GAGTGTGGAGACCATCAAGGAAG |
|  | R, TGCTTTGCGTTGGACATTCAAGTC |
|  | R, GGAAGATGGTGATGGGATT |

**Table S1. Phenotypic characters of the tested onion cultivars**

| **Onion cultivar** | **Phenotypic characters** |  |
| --- | --- | --- |
| **Giza Red (Red onion)** | **The bulbs are solid with dark red cohesive peels and dark red flesh.** | 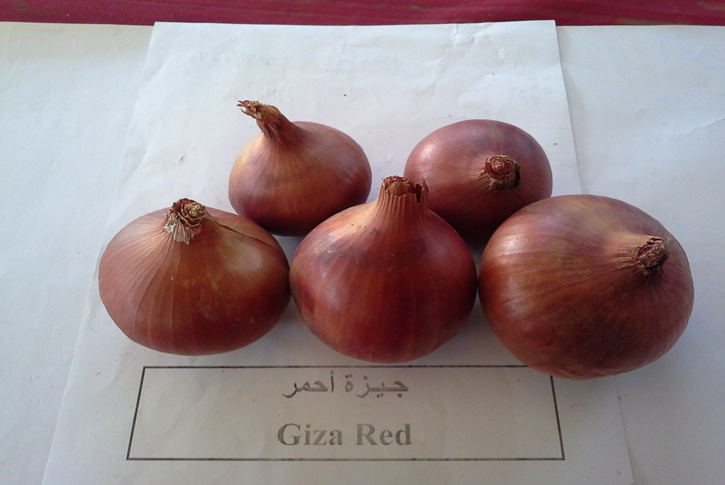 |
| **Giza 6 Mohsen (Golden yellow onion)** | **The bulbs are small, flattened with golden yellow color and thick peels.** | 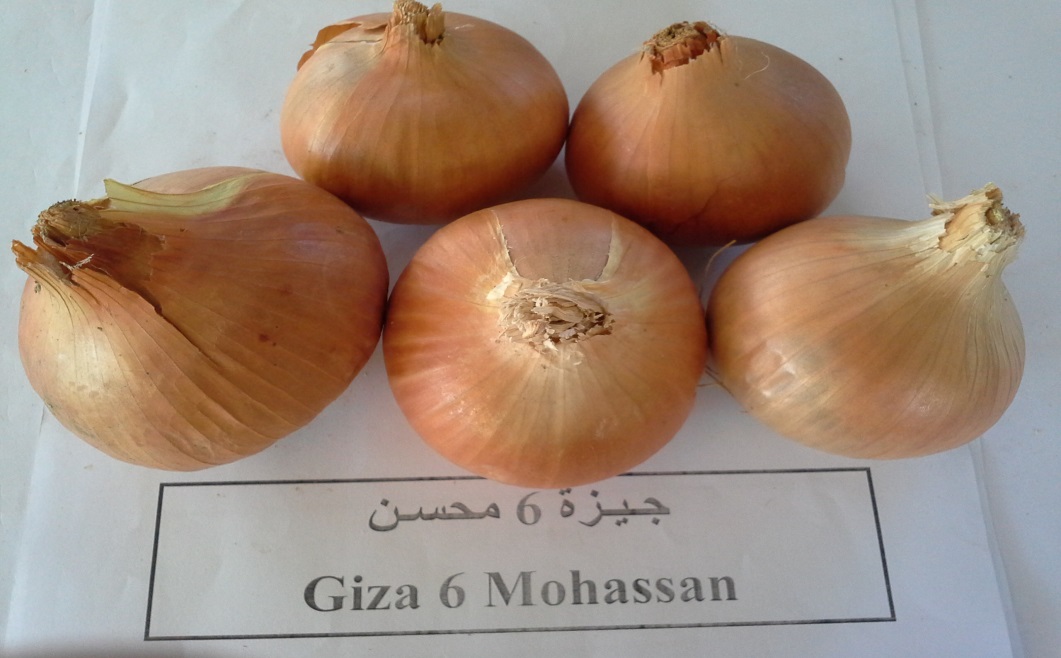 |
| **Giza 20 (Copper-yellow onion)** | **The bulbs are conical in shape with copper-yellow color and thick peels.** | 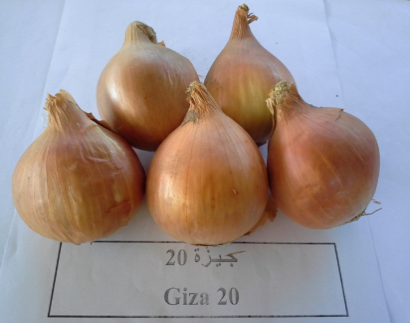 |
| **Giza White (White onion)** | **The bulbs are solid with white, cohesive peels and bright white flesh.** | 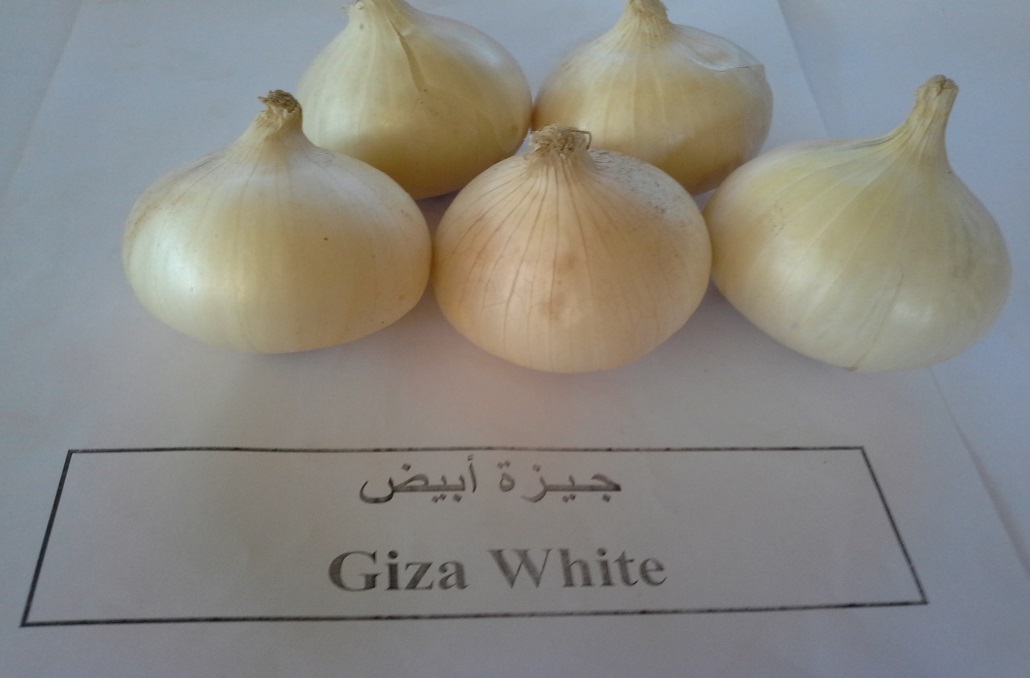 |

**Table S2. Onion samples codes.**

| **Onion sample** | **Code** |
| --- | --- |
| **Red onion peel** | **RP1, RP2 & RP3** |
| **Red onion root** | **RRO1, RRO2 & RRO3** |
| **Golden yellow onion peel** | **GYP1, GYP2 & GYP3** |
| **Golden yellow onion root** | **GYRO1, GYRO2 & GYRO3** |
| **Copper-yellow onion peel** | **CYP1, CYP2 & CYP3** |
| **Copper-yellow onion root** | **CYRO1, CYRO2 & CYRO3** |
| **White onion peel** | **WP1, WP2 & WP3** |
| **White onion root** | **WRO1, WRO2 & WRO3** |

**Table S3. Linearity and sensitivity parameters for alliin, ferulic acid, quercetin, *β*-chlorogenin and palmitic aid**

| **Compound** | **Linearity range (mg /mL)** | **Slope (a)** | **Intercept (b)** | **Correlation coefficient r** | **LOD (mg /mL)** | **LOQ (mg /mL)** |
| --- | --- | --- | --- | --- | --- | --- |
| **Alliin** | **0.015-0.471** | **4.23*10^7^** | **-8.22*10^3^** | **0.992** | **0.009** | **0.017** |
| **Ferulic acid** | **0.021-0.593** | **1.35*10^7^** | **-6.83*10^4^** | **0.997** | **0.01** | **0.012** |
| **Quercetin** | **0.011-0.37** | **2.17*10^7^** | **-5.89*10^4^** | **0.993** | **0.013** | **0.018** |
| ***β*-chlorogenin** | **0.0185-0.61** | **1.49*10^7^** | **-7.43*10^4^** | **0.996** | **0.014** | **0.016** |
| **Palmitic aid** | **0.029-0.57** | **3.11*10^7^** | **-7.55*10^4^** | **0.995** | **0.012** | **0.025** |

**Experimental conditions as in Section 2.3 For each calibration curve the equation is y= ax + b, where y is the peak area, x is the concentration of the standard (mg/mL), a is the slope, b is the intercept, r the correlation coefﬁcient, LOD is the limit of detection and LOQ is the limit of quantitation.**

**Table S4. UPLC-MS metabolite profiling data from onion peels and roots samples representing the content of each variable (peak areas) calculated as (expressed as mg standard equivalents/g dry extract)**

|  | **Peels** | | | | | **Roots** | | | **Reference** | |  |
| --- | --- | --- | --- | --- | --- | --- | --- | --- | --- | --- | --- |
| **ID** | **Name** | **Red onion** | **Copper-yellow onion** | **Golden yellow onion** | **White onion** | **Red onion** | **Copper-yellow onion** | **Golden yellow onion** | **White onion** |  | |
| **1** | ***Alanine** | **0.148 ± 0.005** | **0.176 ± 0.0012** | **0.144 ± 0.002** | **0.157 ± 0.003** | **0.018 ± 0.001** | **0.018 ± 0.0011** | **0** | **0** | [6] | |
| **2** | ***Arginine** | **0** | **0** | **0** | **0** | **0.019 ± 0.002** | **0.018 ± 0.0013** | **0.017 ± 0.0012** | **0.128 ± 0.002** | [6] | |
| **3** | ***Propiin** | **0** | **0** | **0** | **0** | **0.139 ± 0.0021** | **0.214 ± 0.011** | **0.142 ± 0.0012** | **0.133 ± 0.002** | [7] | |
| **4** | ***Methionine** | **0.108 ± 0.001** | **0** | **0** | **0.089 ± 0.003** | **0.083 ± 0.0021** | **0.083 ± 0.001** | **0.072 ± 0.002** | **0** | [6] | |
| **5** | ***Ornithine** | **0.875 ± 0.003** | **0.27 ± 0.001** | **0.265 ± 0.011** | **0.329 ± 0.032** | **0.628 ± 0.021** | **0.761 ±** | **0.631 ±** | **0.313 ±** | [6] | |
| **6** | ***γ -Glutamyl-methionine sulfoxide** | **0.295 ± 0.014** | **0** | **0.013 ± 0.002** | **0.011 ± 0.0001** | **0.085 ± 0.0021** | **0.112 ± 0.0012** | **0.076 ± 0.001** | **0.194 ± 0.0021** | [7] | |
| **7** | ***γ -Glutamyl-glutamine** | **0.435 ± 0.0023** | **0** | **0** | **0.066 ± 0.002** | **0.188 ± 0.0012** | **0.198 ± 0.014** | **0.214 ± 0.012** | **0.229 ± 0.001** | [7] | |
| **8** | ***(Iso)alliin** | **0.036 ± 0.002** | **0.122 ± 0.0021** | **0.07 ± 0.001** | **0.056 ± 0.002** | **0.082 ± 0.001** | **0.137 ± 0.011** | **0.088 ± 0.001** | **0.1 ± 0.002** | [7] | |
| **9** | ****Oxalic acid** | **2.52 ± 0.05** | **11.758 ± 0.044** | **3.616 ± 0.025** | **1.526 ± 0.023** | **0.453 ± 0.002** | **1.117 ± 0.012** | **0.529 ± 0.004** | **0.235 ± 0.0021** | [8] | |
| **10** | ***Allicin** | **0.11 ± 0.0021** | **0.122 ± 0.003** | **0.198 ± 0.0021** | **0.357 ± 0.005** | **0.355 ± 0.001** | **0.432 ± 0.022** | **0.329 ± 0.002** | **0.329 ± 0.013** | [7] | |
| **11** | ****Tartaric acid** | **0** | **0** | **0** | **0.265 ± 0.002** | **0.186 ± 0.02** | **0** | **0** | **0.504 ± 0.015** | [9] | |
| **12** | ****Caffeic acid** | **0** | **0** | **0** | **0.386 ± 0.021** | **0** | **0** | **0** | **0** | [9] | |
| **13** | **** Caffeoylquinic acid** | **0** | **0** | **0** | **0** | **0.256 ± 0.001** | **0.301 ± 0.004** | **0.223 ± 0.002** | **0** | [9] | |
| **14** | ***γ -Glutamyl-*S*-(prop- 2-enyl) cysteine sulfoxide** | **0** | **0** | **0.034 ± 0.001** | **0** | **0.024 ± 0.0051** | **0.032 ± 0.004** | **0.044 ± 0.0012** | **0.023 ± 0.002** | [7] | |
| **15** | ****Feruloylquinic acid** | **0** | **0** | **0** | **0** | **0** | **0.247 ± 0.02** | **0** | **0** | [9] | |
| **16** | ****Malic acid** | **1.842 ± 0.021** | **1.754 ± 0.003** | **1.862 ± 0.015** | **0.437 ± 0.021** | **0** | **0.871 ± 0.002** | **1.546 ± 0.041** | **3.007 ± 0.023** | [10] | |
| **17** | ****Lunularic acid** | **0.483 ± 0.03** | **0** | **0.29 ± 0.002** | **0.145 ± 0.0021** | **1.49 ± 0.002** | **2.053 ± 0.014** | **0.812 ± 0.0012** | **0.22 ± 0.003** | [7] | |
| **18** | ***Pro Betaine (*N, N*-Dimethyl-Proline)** | **0** | **0** | **0** | **0** | **0.438 ± 0.021** | **0.243 ± 0.003** | **0.408 ± 0.0012** | **0.207 ± 0.0018** | [7] | |
| **19** | ***Glutamic acid** | **0** | **0** | **0.191 ± 0.002** | **0.34 ± 0.0012** | **0** | **0.18 ± 0.001** | **0** | **0.103 ± 0.0021** | [6] | |
| **20** | ***Pipecolic acid** | **0.246 ± 0.021** | **0** | **0** | **0.116 ± 0.002** | **0.071 ± 0.001** | **0.078 ± 0.0023** | **0.042 ± 0.002** | **0.049 ± 0.001** | [7] | |
| **21** | ****S*-Methyl methionine** | **0.15 ± 0.002** | **0.125 ± 0.003** | **0.066 ± 0.002** | **0.171 ± 0.0022** | **0.061 ± 0.0021** | **0** | **0.051 0.0012** | **0.146 0.002** | [7] | |
| **22** | ****Citric acid** | **0.228 ± 0.003** | **0.618 ± 0.002** | **0.428 ± 0.002** | **0** | **0.235 ± 0.002** | **0.246 ± 0.001** | **0.207 ± 0.021** | **1.107 ± 0.01** | [10] | |
| **23** | ****Vanillic acid** | **0.234 ± 0.003** | **0** | **0.28 ± 0.001** | **0** | **0** | **0** | **0** | **0** | [11] | |
| **24** | ***Pyroglutamic acid** | **0.037 ± 0.001** | **0.056 ± 0.0031** | **0.068 ± 0.003** | **0.131 ± 0.002** | **0.022 ± 0.001** | **0.191 ± 0.002** | **0.023 ± 0.003** | **0.103 ± 0.0012** | [6] | |
| **25** | ****Rosmarinic acid** | **0.362 ± 0.031** | **0** | **0.422 ± 0.002** | **0.271 ± 0.0023** | **0** | **0** | **0** | **0** | [10] | |
| **26** | ***γ -Glutamyl-*S*-(2-carboxypropyl) cysteine-glycine** | **0** | **0** | **0.03 ± 0.001** | **0.047 ± 0.002** | **0** | **0.051 ± 0.002** | **0.023 ± 0.0011** | **0.103 ± 0.001** | [7] | |
| **27** | ***γ -Glutamyl-methionine** | **0** | **0** | **0** | **0** | **0** | **0.077 ± 0.002** | **0** | **0** | [7] | |
| **28** | ***** Cyanidin 3-*O*-(malonyl-acetyl)-glucoside** | **0.14± 0.001** | **0** | **0** | **0** | **0.677 ± 0.0012** | **0** | **0.435 ± 0.002** | **0.124 ± 0.02** | [12] | |
| **29** | ****Glycolic acid** | **0.449 ± 0.002** | **0.885 ± 0.003** | **0.637 ± 0.0012** | **0.433 ± 0.002** | **0.586 ± 0.02** | **1.004 ± 0.03** | **0.558 ± 0.002** | **0.276 ± 0.021** | [8] | |
| **30** | ***γ -Glutamyl-*S*-(propyl) cysteine sulfoxide** | **0.583 ± 0.001** | **0.711 ± 0.01** | **0.724 ± 0.023** | **0.174 ± 0.021** | **0.353 ± 0.001** | **0.428 ± 0.02** | **0.2 ± 0.001** | **0.064 ± 0.002** | [7] | |
| **31** | ****succinic acid** | **0.176 ± 0.001** | **0** | **0** | **0** | **0** | **0** | **0** | **0** | [8] | |
| **32** | ****Ferulic acid** | **0** | **0** | **0** | **0** | **0** | **0** | **0** | **0.381 ± 0.002** | [9] | |
| **33** | ****S*-(prop-1-enyl) cysteine sulfoxide - *S*-(prop-1-enyl) cysteine sulfoxide** | **0.071 ± 0.001** | **0** | **0.103 ± 0.001** | **0** | **0** | **0** | **0** | **0.071 ± 0.002** | [7] | |
| **34** | ****Ascorbic acid** | **0.645 ± 0.001** | **1.011 ± 0.03** | **0.39 ± 0.02** | **0** | **0** | **0.371 ± 0.001** | **0** | **0** | [13] | |
| **35** | ***Tryptophan** | **0** | **0** | **0** | **0** | **0** | **0** | **0** | **0.015 ± 0.002** | [6] | |
| **36** | ****Sinapic acid** | **0** | **0** | **0** | **0.15 ± 0.02** | **0** | **0** | **0** | **0.209 ± 0.01** | [14] | |
| **37** | *****p*-Coumaroyl glycolic acid** | **0** | **0.223 ± 0.001** | **0** | **0** | **0** | **0** | **0** | **0** | [15] | |
| **38** | ****Hydroxytyrosol** | **0.903 ± 0.012** | **0.896 ± 0.03** | **0.88 ± 0.021** | **0.176 ± 0.02** | **1.559 ± 0.021** | **0.817 ± 0.01** | **0.47 ± 0.001** | **0** | [16] | |
| **39** | ***Asparagine** | **0** | **0** | **0** | **0** | **0** | **0.172 ± 0.002** | **0** | **0.063 ± 0.001** | [6] | |
| **40** | ****Trihydroxyphenylglyoxylate** | **1.362 ± 0.02** | **0** | **0.95 ± 0.02** | **0** | **0** | **0** | **0** | **0** | [17] | |
| **41** | ****3'-Methoxylunularic acid** | **0** | **0.465 ± 0.003** | **0.32 ± 0.001** | **0** | **0.294 ± 0.003** | **0.286 ± 0.002** | **0.257 ± 0.02** | **0** | [7] | |
| **42** | ****Lunularin 4-*O*-hexoside** | **0.354 ± 0.002** | **0** | **0** | **0.229 ± 0.01** | **0.446 ± 0.003** | **0.373 ± 0.001** | **0** | **0.354 ± 0.02** | [7] | |
| **43** | ***** Epigallocatechin** | **0** | **0** | **0** | **0** | **0.187 ± 0.021** | **0** | **0** | **0.629 ± 0.02** | [18] | |
| **44** | ***3-methoxytyramine** | **0.144 ± 0.001** | **0** | **0** | **0.071 ± 0.0021** | **0** | **0** | **0** | **0** | [7] | |
| **45** | ***** Apigenin *O*-pentosyl-hexoside** | **0.377 ± 0.0021** | **0.895 ± 0.01** | **0** | **0.233 ± 0.021** | **0.384 ± 0.023** | **0.155 ± 0.002** | **0** | **0.275 ± 0.02** | [19] | |
| **46** | ***γ -Glutamyl *S*-(prop-1-enyl) cysteine** | **0.277 ± 0.01** | **0.179 ± 0.003** | **0.177 ± 0.002** | **0** | **0** | **0** | **0** | **0** | [7] | |
| **47** | *****Cyanidin 3-*O*-acetylglucoside** | **0.657 ± 0.002** | **0.459 ± 0.0023** | **0.586 ± 0.01** | **0** | **0.166 ± 0.011** | **0.132 ± 0.021** | **0.127 ± 0.001** | **0.129 ± 0.002** | [12, 20] | |
| **48** | *****Cyanidin 3-*O*-malonylglucoside** | **0.251 ± 0.02** | **0** | **0** | **0** | **0** | **0** | **0** | **0** | [12] | |
| **49** | ******Tropeoside B** | **0.946 ± 0.003** | **0** | **0** | **0** | **0** | **0** | **0** | **0** | [21] | |
| **50** | *****Peonidin 3-*O*-glucoside** | **0.127 ± 0.001** | **0** | **0** | **0** | **0.115 ± 0.02** | **0** | **0** | **0.088 ± 0.012** | [12] | |
| **51** | *****Quercetin 3,4'-*O*-diglucoside** | **0.504 ± 0.021** | **1.635 ± 0.012** | **0.444 ± 0.02** | **0** | **2.408 ± 0.031** | **1.777 ± 0.023** | **1.693 ± 0.001** | **1.269 ± 0.021** | [20, 22] | |
| **52** | *****Isorhamnetin 3,4'-*O* diglucoside** | **1.271 ± 0.021** | **0.492 ± 0.0023** | **0.483 ± 0.021** | **0** | **0.488 ± 0.001** | **0** | **0.132 ± 0.011** | **0.172 ± 0.003** | [20] | |
| **53** | *****2-(3,4-Dihydroxybenzoyl)-2,4,6- trihydroxy-3(2H)-benzofuranone** | **1.535 ± 0.011** | **1.343 ± 0.02** | **1.484 ± 0.002** | **0** | **0.243 ± 0.002** | **0.321 ± 0.003** | **0.238 ± 0.005** | **0.183 ± 0.001** | [22, 23] | |
| **54** | ******Ascalonicoside A** | **0.591 ± 0.02** | **0** | **2.954 ± 0.02** | **0** | **0.967 ± 0.031** | **1.097 ± 0.021** | **1.245 ± 0.04** | **1.224 ±0.021** | [21] | |
| **55** | *****Dihydroquercetin 3-*O*-rhamnoside** | **0** | **0** | **0** | **0** | **0** | **0** | **0** | **0.065** | [24] | |
| **56** | *****kaempferol *O*-rhamnosyl-hexoside** | **0.464 ± 0.021** | **0.21 ± 0.001** | **0.207 ± 0.003** | **0** | **0.264 ± 0.001** | **0.199 ± 0.012** | **0.16 ± 0.002** | **0.094 ± 0.003** | [25] | |
| **57** | ******Ceposide A** | **11.428 ± 0.023** | **9.587 ± 0.014** | **11.396 ± 0.032** | **0** | **5.457 ± 0.012** | **2.942 ± 0.013** | **3.025 ± 0.001** | **2.783 ± 0.0021** | [21] | |
| **58** | *****Quercetin 3-*O*-glucoside** | **0.646 ± 0.021** | **0.085 ± 0.001** | **0.521 ± 0.002** | **0** | **0.23 ± 0.001** | **0.256 ± 0.0021** | **0.255 ± 0.003** | **0.247 ± 0.001** | [20] | |
| **59** | *****Quercetin 4'-*O*-glucoside** | **0.693 ± 0.02** | **4.272 ± 0.023** | **0.687 ± 0.021** | **0.32 ± 0.002** | **0.426 ± 0.021** | **0.654 ± 0.002** | **0.149 ± 0.003** | **0.2 ± 0.0012** | [20] | |
| **60** | ******Ceposide C** | **0.3 ± 0.001** | **0.276 ± 0.0021** | **0** | **0** | **0** | **0** | **0** | **0** | [21] | |
| **61** | *****Isorhamnetin-3-*O*-glucoside** | **2.173 ± 0.002** | **1.039 ± 0.003** | **2.013 ± 0.001** | **0** | **2.556 ± 0.012** | **2.072 ± 0.051** | **2.528 ± 0.042** | **2.324 ± 0.024** | [20] | |
| **62** | *****Isorhamnetin 4'-*O*-glucoside** | **0** | **0.063 ± 0.001** | **0** | **0** | **0** | **0** | **0** | **0** | [26] | |
| **63** | *****Adduct of quercetin with protocatechuic acid** | **0.4 ± 0.002** | **0.521 ± 0.003** | **1.054 ± 0.0012** | **0** | **1.902 ± 0.02** | **2.508 ± 0.032** | **2.592 ± 0.001** | **1.788 ± 0.021** | [23] | |
| **64** | ***** Quercetin dimer hexoside** | **0.286 ± 0.001** | **0** | **0** | **0** | **0.143 ± 0.02** | **0.174 ± 0.003** | **0.241 ± 0.002** | **0.104 ± 0.012** | [23] | |
| **65** | *****N*-(*p*-Coumaroyl)-tyramine** | **0** | **0** | **0.245 ± 0.03** | **0.252 ± 0.002** | **3.012 ± 0.014** | **3.025 ± 0.001** | **2.279 ± 0.021** | **1.065 ± 0.001** | [21] | |
| **66** | *****Hesperetin** | **11.012 ± 0.04** | **6.404 ± 0.05** | **0.843 ± 0.002** | **0** | **0** | **0** | **0** | **0** | [27] | |
| **67** | *****N*-Feruloyl-tyramine** | **1.994 ± 0.023** | **2.923 ± 0.021** | **2.648 ± 0.002** | **0.291 ± 0.021** | **3.664 ± 0.01** | **4.057 ± 0.003** | **3.495 ± 0.002** | **2.827 ± 0.02** | [7] | |
| **68** | ******Ceparocide I** | **1.632 ± 0.03** | **1.769 ± 0.002** | **1.619 ± 0.02** | **0** | **4.422 ± 0.021** | **5.956 ± 0.002** | **6.333 ± 0.03** | **4.298 ± 0.021** | [21] | |
| **69** | ***Allylmercaptoglutathione** | **0** | **0** | **0** | **0** | **0.222 ± 0.001** | **0.146 ± 0.003** | **0.109 ± 0.021** | **0.091 ± 0.002** | [7] | |
| **70** | *****N*-Feruloyl-3-methoxytyramine** | **0** | **0** | **0** | **0** | **1.147 ± 0.001** | **0.786 ± 0.032** | **0.758 ± 0.012** | **0.419 ± 0.001** | [7] | |
| **71** | *****Adduct of quercetin with methyl protocatechuate** | **0.138 ± 0.012** | **0.195 ± 0.002** | **0.279 ± 0.004** | **0.139 ± 0.001** | **1.093 ± 0.0021** | **1.339 ± 0.002** | **1.652 ± 0.012** | **1.087 ± 0.023** | [23] | |
| **72** | *****Morin** | **2.972 ± 0.012** | **1.851 ± 0.002** | **1.552 ± 0.021** | **0.45 ± 0.01** | **3.565 ± 0.012** | **1.965 ± 0.023** | **2.459 ± 0.03** | **0** | [28] | |
| **73** | *****Quercetin** | **2.163 ± 0.021** | **1.051 ± 0.021** | **1.347 ± 0.002** | **0** | **0.85 ± 0.022** | **0.184 ± 0.023** | **0** | **0.136 ± 0.021** | [23] | |
| **74** | ******Tropeoside A methyl derivative** | **0.45 ± 0.001** | **0.382 ± 0.024** | **0.659 ± 0.001** | **0.886 ± 0.021** | **0.449 ± 0.003** | **0.712 ± 0.005** | **0.584 ± 0.002** | **0.313 ± 0.001** | [21] | |
| **75** | *****3-(Quercetin-8-yl)-2,3-epoxyflavanone** | **1.31 ± 0.002** | **0** | **1.366 ± 0.031** | **0.106 ± 0.01** | **0.531 ± 0.003** | **1.157 ± 0.012** | **0.768 ± 0.002** | **0.333 ± 0.001** | [23] | |
| **76** | *****Taxifolin** | **0** | **0** | **1.521 ± 0.002** | **0** | **0.241 ± 0.011** | **0.275 ± 0.002** | **0.204 ± 0.005** | **0.229 ± 0.001** | [29] | |
| **77** | *****3'-Hydroxymelanettin** | **0.216 ± 0.002** | **0** | **0.376 ± 0.01** | **0** | **0** | **0** | **0** | **0** | [30] | |
| **78** | ******Dihydroxypegnadienone *O*- rhamnosyl-pentoside** | **0** | **0** | **0** | **0** | **0.399 ± 0.005** | **0.471 ± 0.04** | **0.435 ± 0.02** | **0.258 ± 0.012** | [21] | |
| **79** | ***** Quercetin dimer hexoside isomer** | **1.572 ± 0.001** | **1.353 ± 0.01** | **0.63 ± 0.002** | **0** | **0.213 ± 0.001** | **0** | **0** | **0** | [23] | |
| **80** | *****Sativanone** | **0.216 ± 0.01** | **0** | **0** | **0** | **0** | **0** | **0** | **0** | [30] | |
| **81** | *****Kaempferol** | **3.475 ± 0.002** | **2.835 ± 0.001** | **1.829 ± 0.02** | **0.009 ± 0.001** | **3.279 ± 0.0021** | **0.402 ± 0.05** | **0.273 ± 0.031** | **0** | [23] | |
| **82** | ****** Trihydroxyspirosten *O*- rhamnosyl-pentoside** | **0.696 ± 0.01** | **0** | **4.023 ± 0.02** | **0** | **2.854 ± 0.003** | **3.629 ± 0.02** | **2.829 ± 0.01** | **0.606 ± 0.002** | [21] | |
| **83** | *****Isorhamnetin** | **1.36 ± 0.021** | **1.318 ± 0.001** | **1.521 ± 0.05** | **0.093 ± 0.001** | **2.085 ± 0.03** | **1.139 ± 0.021** | **0.934 ± 0.002** | **0.771 ± 0.005** | [31] | |
| **84** | *****Phloroglucinoyl-dihydroxybenzoate** | **0.082 ± 0.001** | **0** | **0.087 ± 0.02** | **0** | **0** | **0** | **0** | **0** | [17] | |
| **85** | ******Randiasaponin IV** | **0** | **0** | **0** | **0** | **0** | **0.239 ± 0.01** | **0** | **0** | [21] | |
| **86** | *****Quercetin dimer** | **2.311 ± 0.05** | **0.371 ± 0.001** | **2.6 ± 0.02** | **0** | **0.763 ± 0.015** | **0.616 ± 0.02** | **0.536 ± 0.05** | **0** | [23] | |
| **87** | *****Trihydroxy- methoxyisoflavanone** | **0.136 ± 0.021** | **0.085 ± 0.001** | **0.144 ± 0.01** | **0.149 ± 0.001** | **0.047 ± 0.003** | **0.037 ± 0.01** | **0.043 ± 0.005** | **0** | [30, 32] | |
| **88** | *****Adduct of quercetin dimer with methyl protocatechuate** | **1.168 ± 0.002** | **0.307 ± 0.01** | **1.626 ± 0.023** | **0** | **0** | **0** | **0** | **0** | [23] | |
| **89** | *****Quercetin trimer** | **3.635 ± 0.021** | **1.552 ± 0.001** | **1.885 ± 0.05** | **0** | **2.168 ± 0.02** | **0** | **0.928 ± 0.052** | **0** | [23] | |
| **90** | *****Dihydroxy-dimethoxyisoflavone** | **0** | **0** | **0.184 ± 0.021** | **0** | **0.176 ± 0.002** | **0.126 ± 0.05** | **0.146 ± 0.023** | **0.112 ± 0.002** | [30] | |
| **91** | ******Alliospiroside D** | **5.283 ± 0.015** | **6.292 ± 0.013** | **1.861 ± 0.05** | **0** | **1.045 ± 0.011** | **0.808 ± 0.021** | **0.945 ± 0.011** | **0.966 ± 0.03** | [21] | |
| **92** | ******Alliospiroside B** | **0.837 ± 0.004** | **0** | **0.374 ± 0.07** | **0.676 ± 0.021** | **4.478 ± 0.015** | **5.003 ± 0.002** | **4.768 ± 0.01** | **3.226 ± 0.021** | [21] | |
| **93** | *****Naringenin** | **0.54 ± 0.001** | **0.941 ± 0.0023** | **4.933 ± 0.002** | **2.246 ± 0.001** | **8.845 ± 0.014** | **9.655 ± 0.002** | **6.943 ± 0.05** | **5.283 ± 0.016** | [27] | |
| **94** | ******Alliospiroside A** | **0.296 ± 0.05** | **0.433 ± 0.022** | **0.502 ± 0.001** | **0.765 ± 0.02** | **3.618 ± 0.003** | **4.277 ± 0.002** | **2.954 ± 0.051** | **1.896 ± 0.02** | [21] | |
| **95** | ******* Oxo-octadecenoic acid** | **0** | **0** | **0** | **0** | **0** | **1.053 ± 0.02** | **0.146 ± 0.005** | **0** | [33] | |
| **96** | ******* Myristic acid** | **0.122 ± 0.001** | **0** | **0.092 ± 0.002** | **0** | **0** | **0** | **0** | **0** | [33] | |
| **97** | ******* Palmitic acid** | **0** | **0** | **0** | **0** | **0.119 ± 0.01** | **0.144 ± 0.02** | **0** | **0** | [33] | |
| **98** | ******* Stearic acid** | **0.349 ± 0.05** | **0** | **0** | **0.069 ± 0.002** | **0** | **0** | **0** | **0** | [33] | |
| **99** | *******β*-Chlorogenin** | **1.233 ± 0.001** | **1.401 ± 0.002** | **1.4 ± 0.05** | **0** | **0** | **0** | **0** | **0** | [33] | |
| **100** | ******* Oleic acid** | **0** | **0** | **0.114 ± 0.001** | **0.1 ± 0.015** | **0.024 ± 0.002** | **0** | **0.053 ± 0.02** | **0.07 ± 0.005** | [33] | |
| **101** | ******* Gadooleic acid** | **0** | **0** | **0** | **0** | **0** | **0** | **0** | **0.104 ± 0.05** | [33] | |
| **102** | ******* Linolenic acid** | **0** | **0** | **0** | **0** | **0.114 ± 0.05** | **0.143 ± 0.003** | **0.11 ± 0.012** | **0** | [33] | |
| **103** | ******Diosgenin** | **0** | **0** | **0** | **0.222** **± 0.002** | **0** | **0** | **0.283 ± 0.04** | **0** | [34] | |

**Data are expressed as the average of three determinations ± SD**

*** Compounds are expressed** **as alliin equivalent**

**** Compounds are expressed as ferulic acid equivalent**

******* **Compounds are expressed as quercetin** **equivalent**

****** Compounds are expressed as *β*-chlorogenin equivalent**

******* Compounds are expressed as palmitic acid equivalent**

**Table S5. Performance parameters for the PLSR models created for PDE-5 inhibitory and anti-inflammatory activities.**

|  |  | **LVs** | **Calibration** | | **Cross-validation** | | **Test (prediction)** | |
| --- | --- | --- | --- | --- | --- | --- | --- | --- |
|  |  |  | **R^2^** | **RMSEE** | **R^2^** | **RMSECV** | **R^2^** | **RMSEP** |
| **PDE-5 inhibitory activity** |  | 4 | 0.9915 | 0.00257 | 0.982 | 0.00605 | 0.9917 | 0.00279 |
| **Anti-inflammatory activity** | **IL-1*β*** | 4 | 0.959 | 0.108 | 0.961 | 0.136 | 0.982 | 0.075 |
|  | **IL-6** | 4 | 0.984 | 0.087 | 0.961 | 0.119 | 0.969 | 0.113 |
|  | **IFN-*γ*** | 4 | 0.963 | 0.111 | 0.961 | 0.172 | 0.9822 | 0.082 |
|  | **TNF-*α*** | 4 | 0.994 | 0.061 | 0.961 | 0.2 | 0.99 | 0.08 |

**R^2^ = coefficient of determination, RMSEE = Root mean square error of estimation, RMSECV = Root mean square error of cross-validation and** **RMSEP = Root mean square error of prediction.**

**Table S6. Classification parameters using PDE-5 inhibition PLS class model**

| **Model** |  | **Class** | **Specificity** | **Sensitivity** | **Accuracy** | **Efficiency** |
| --- | --- | --- | --- | --- | --- | --- |
| **PLS-Class model** | Training (calibration) | Red peel | 100% | 100% | 100% | 100% |
|  |  | Copper-yellow peel | 100% | 100% | 100% | 100% |
|  |  | Golden yellow peel | 100% | 100% | 100% | 100% |
|  |  | White peel | 100% | 100% | 100% | 100% |
|  |  | Red root | 100% | 100% | 100% | 100% |
|  |  | Copper-yellow root | 100% | 100% | 100% | 100% |
|  |  | Golden yellow root | 100% | 100% | 100% | 100% |
|  |  | White root | 100% | 85% | 93.7% | 92% |
|  | Test  (prediction) | Red peel | 100% | 100% | 100% | 100% |
|  |  | Copper-yellow peel | 100% | 100% | 100% | 100% |
|  |  | Golden yellow peel | 100% | 100% | 100% | 100% |
|  |  | White peel | 100% | 100% | 100% | 100% |
|  |  | Red root | 100% | 100% | 100% | 100% |
|  |  | Copper-yellow root | 100% | 100% | 100% | 100% |
|  |  | Golden yellow root | 100% | 100% | 100% | 100% |
|  |  | White root | 100% | 90% | 96.8% | 94% |

**Table S7. Classification parameters using anti-inflammatory PLS class model**

| **Model** |  | **Class** | **Specificity** | **Sensitivity** | **Accuracy** | **Efficiency** |
| --- | --- | --- | --- | --- | --- | --- |
| **PLS-Class model** | Training (calibration) | Red peel | 100% | 90% | 96.8% | 94% |
|  |  | Copper-yellow peel | 100% | 90% | 96.8% | 94% |
|  |  | Golden yellow peel | 100% | 90% | 96.8% | 94% |
|  |  | White peel | 100% | 100% | 100% | 100% |
|  |  | Red root | 100% | 100% | 100% | 100% |
|  |  | Copper-yellow root | 100% | 85% | 93.7% | 92% |
|  |  | Golden yellow root | 100% | 85% | 93.7% | 92% |
|  |  | White root | 100% | 100% | 100% | 100% |
|  | Test  (prediction) | Red peel | 100% | 90% | 96.8% | 94% |
|  |  | Copper-yellow peel | 100% | 90% | 96.8% | 94% |
|  |  | Golden yellow peel | 100% | 90% | 96.8% | 94% |
|  |  | White peel | 100% | 100% | 100% | 100% |
|  |  | Red root | 100% | 100% | 100% | 100% |
|  |  | Copper-yellow root | 100% | 85% | 93.7% | 92% |
|  |  | Golden yellow root | 100% | 85% | 93.7% | 92% |
|  |  | White root | 100% | 100% | 100% | 100% |


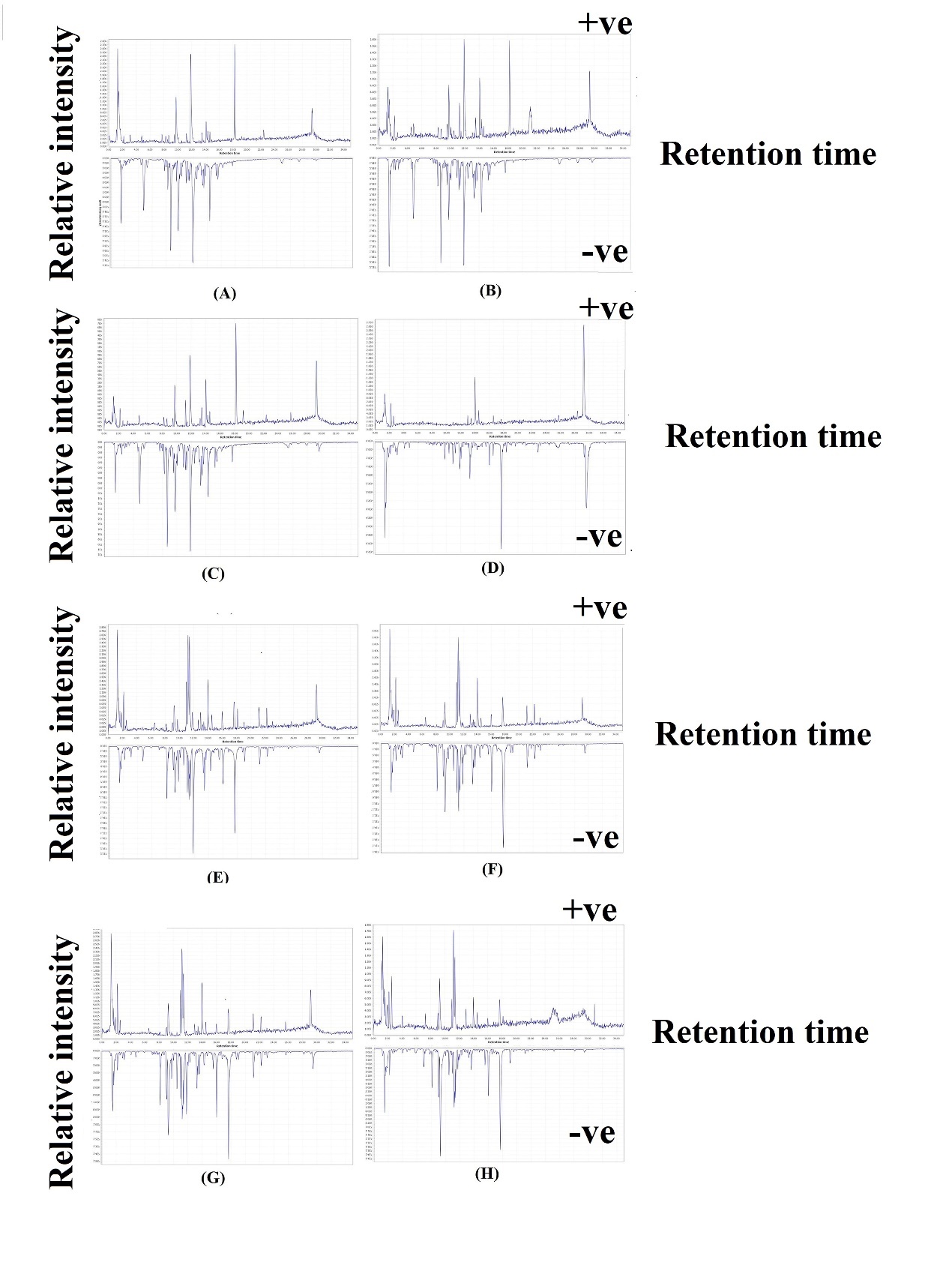


**Figure S1. Base peak chromatograms (BPC) collected in the negative and positive modes for peel and root extracts of the four onion cultivars: (A) Red onion peel, (B)** **Copper-yellow onion peel, (C) Golden yellow onion peel, (D) White onion peel, (E) Red onion root, (F) Copper-yellow onion root, (G) Golden yellow onion root, (H) White onion root.**


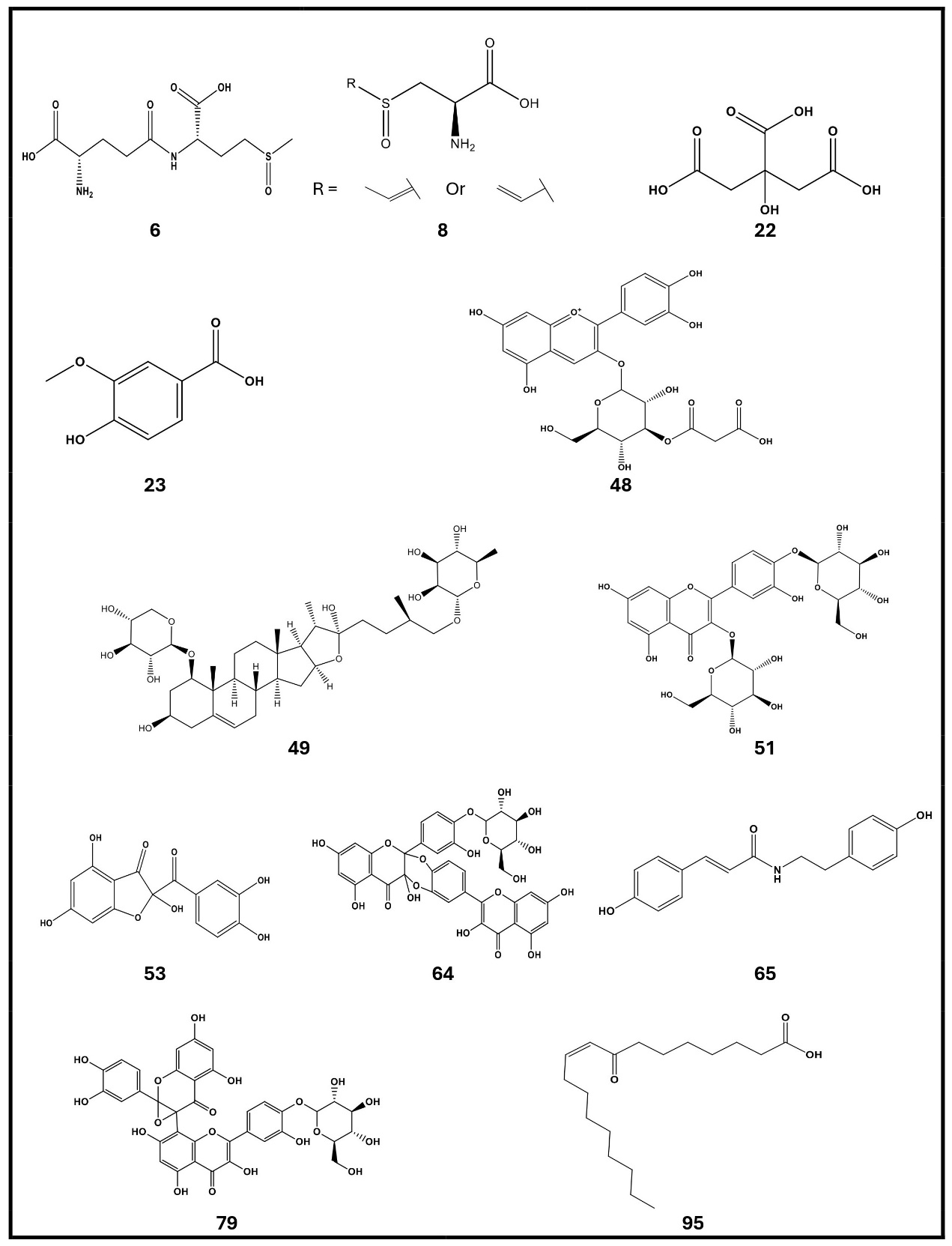


**Figure S2. Chemical structures of the discussed compounds.**


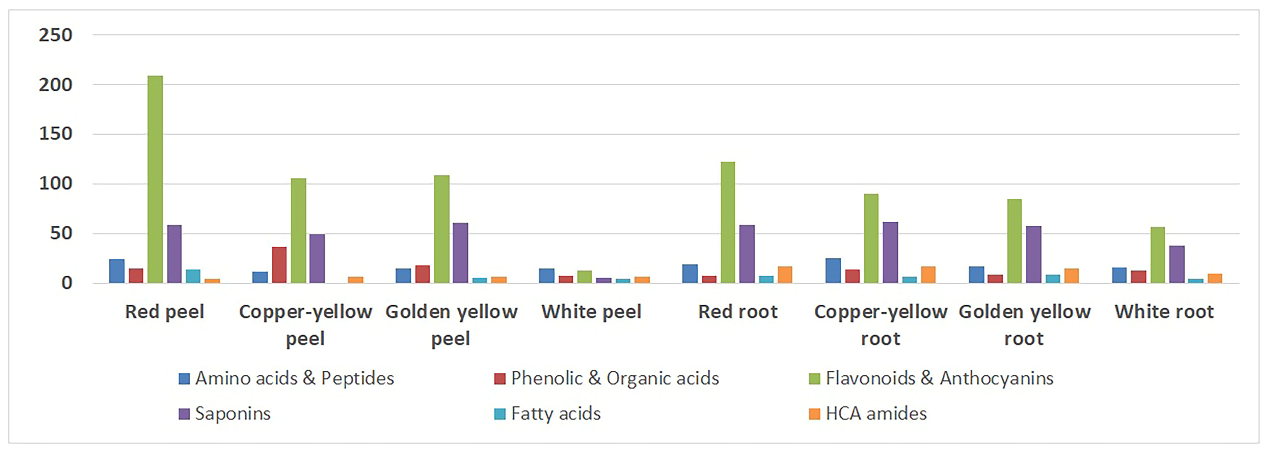


**Figure S3. Bar chart demonstrating relative quantitation of the total content of different metabolite classes identified in peel and root extracts of the tested onion cultivars expressed as mg equivalents per 100 g dry weight.**


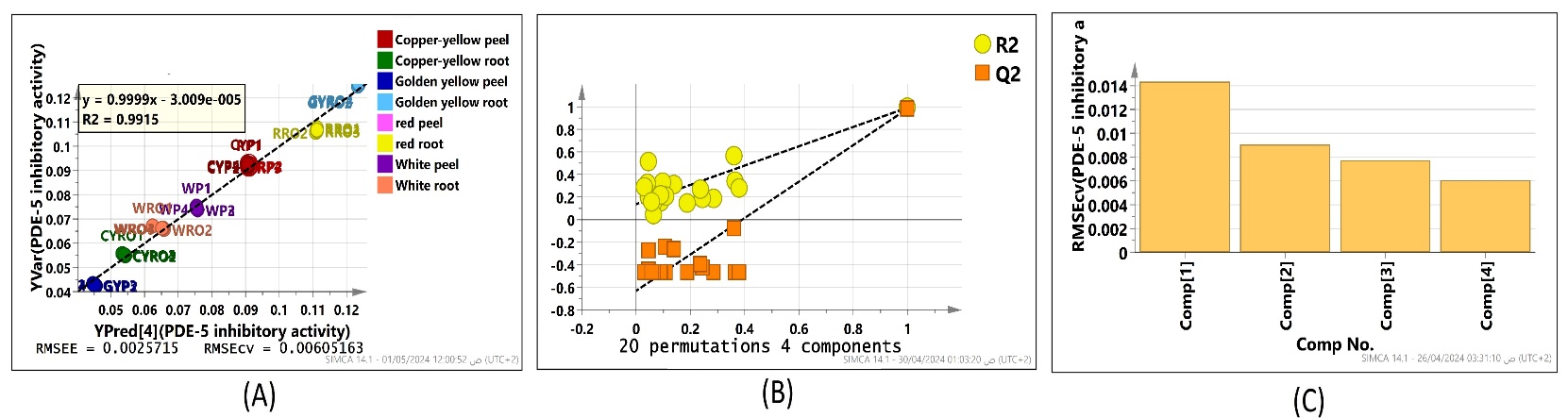


**Figure S4. Validation for PLSR model of PDE-5 inhibitory activity exerted by root and peel samples of the tested onion cultivars. (A) the relationship between observed and predicted values, (B) permutation plots, (C) correlation between RMSECV and PLS optimal latent variables,** **RMSEE = Root mean square error of estimation and RMSECV = Root mean square error of cross-validation.**


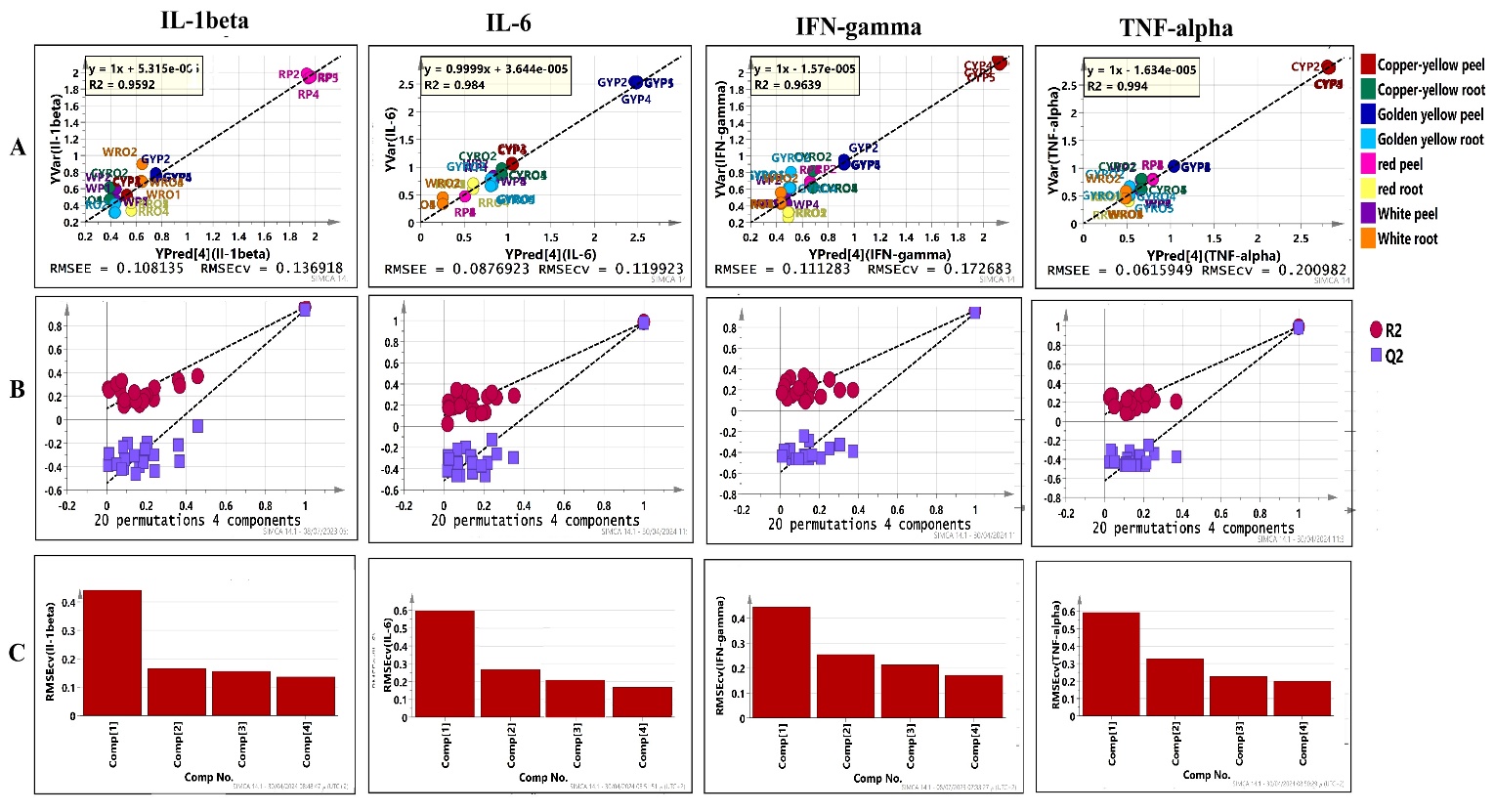
**Figure S5. Validation for PLSR model of pro-inflammatory markers inhibition displayed by root and peel samples of the tested onion cultivars. (A) the relationship between observed and predicted values, (B) permutation plots, (C) correlation between RMSECV and PLS optimal latent variables, RMSEE = Root mean square error of estimation and RMSECV = Root mean square error of cross-validation.**

**References**

1. Kadian N, Raju KSR, Rashid M, Malik MY, Taneja I, Wahajuddin M. Comparative assessment of bioanalytical method validation guidelines for pharmaceutical industry. J Pharm Biomed Anal. 2016;126:83–97.

2. Kelly SJ, Butler LG. Enzymic Hydrolysis of Phosphonate Esters. Reaction Mechanism of Intestinal 5′-Nucleotide Phosphodiesterase. Biochemistry. 1977;16:1102–4.

3. Oboh G, Adebayo AA, Ademosun AO, Boligon AA. In vitro inhibition of phosphodiesterase-5 and arginase activities from rat penile tissue by two Nigerian herbs (Hunteria umbellata and Anogeissus leiocarpus). J Basic Clin Physiol Pharmacol. 2017;28:393–401.

4. Mosmann T. Rapid Colorimetric Assay for Cellular Growth and Survival: Application to Proliferation and Cytotoxicity Assays. J Immunol Methods. 1983;65:55–63.

5. Louis KS, Siegel AC. Cell Viability Analysis Using Trypan Blue: Manual and Automated Methods. Methods Mol Biol. 2011;740:7–12.

6. Piraud M, Vianey-Saban C, Petritis K, Elfakir C, Steghens J-P, Morla A, et al. ESI-MS/MS analysis of underivatised amino acids: a new tool for the diagnosis of inherited disorders of amino acid metabolism. Fragmentation study of 79 molecules of biological interest in positive and negative ionisation mode. Rapid Commun Mass Spectrom. 2003;17:1297–1311.

7. Böttcher C, Krähmer A, Stürtz M, Widder S, Schulz H. Comprehensive metabolite profiling of onion bulbs (Allium cepa) using liquid chromatography coupled with electrospray ionization quadrupole time-of-flight mass spectrometry. Metabolomics. 2017;13:1–15.

8. Brent LC, Reiner JL, Dickerson RR, Sander LC. Method for characterization of low molecular weight organic acids in atmospheric aerosols using ion chromatography mass spectrometry. Anal Chem. 2014;86:7328–36.

9. Fang N, Yu S, Prior RL. LC/MS/MS Characterization of Phenolic Constituents in Dried Plums. J Agric Food Chem. 2002;50:3579−3585.

10. Hu T, Zheng K, Liang J, Tang D, Zhang L, Xian M, et al. Integrated UHPLC-MS and network pharmacology to explore the active constituents and pharmacological mechanisms of Shenzao dripping pills against coronary heart disease. Tradit Med Res. 2022;7:24.

11. Gruz J, Novák O, Strnad M. Rapid analysis of phenolic acids in beverages by UPLC-MS/MS. Food Chem. 2008;111:789–94.

12. Wu X, Prior RL. Identification and characterization of anthocyanins by high-performance liquid chromatography-electrospray ionization-tandem mass spectrometry in common foods in the United States: Vegetables, nuts, and grains. J Agric Food Chem. 2005;53:3101–13.

13. Garrido Frenich A, Hernández Torres ME, Belmonte Vega A, Martínez Vidal JL, Plaza Bolaños P. Determination of ascorbic acid and carotenoids in food commodities by liquid chromatography with mass spectrometry detection. J Agric Food Chem. 2005;53:7371–6.

14. Narváez-Cuenca CE, Vincken JP, Gruppen H. Identification and quantification of (dihydro) hydroxycinnamic acids and their conjugates in potato by UHPLC-DAD-ESI-MSn. Food Chem. 2012;130:730–8.

15. Ali A, Cottrell JJ, Dunshea FR. Antioxidant, Alpha-Glucosidase Inhibition Activities, In Silico Molecular Docking and Pharmacokinetics Study of Phenolic Compounds from Native Australian Fruits and Spices. Antioxidants. 2023;12:1–27.

16. Mazzotti F, Benabdelkamel H, Di Donna L, Maiuolo L, Napoli A, Sindona G. Assay of tyrosol and hydroxytyrosol in olive oil by tandem mass spectrometry and isotope dilution method. Food Chem. 2012;135:1006–10.

17. Elsadig Karar MG, Kuhnert N. UPLC-ESI-Q-TOF-MS/MS Characterization of Phenolics from Crataegus monogyna and Crataegus laevigata (Hawthorn) Leaves, Fruits and their Herbal Derived Drops (Crataegutt Tropfen). J Chem Biol Ther. 2016;01:1–23.

18. Chua LS, Latiff NA, Lee SY, Lee CT, Sarmidi MR, Aziz RA. Flavonoids and phenolic acids from Labisia pumila (Kacip Fatimah). Food Chem. 2011;127:1186–92.

19. Dias MI, Barros L, Dueñas M, Pereira E, Carvalho AM, Alves RC, et al. Chemical composition of wild and commercial Achillea millefolium L. and bioactivity of the methanolic extract, infusion and decoction. Food Chem. 2013;141:4152–60.

20. Vijayalakshmi G, Raja MM, Naik ML, Carbone V, Russo GL, Khan PSSV. Determination of antioxidant capacity and flavonoid composition of onion (Allium cepa L.) landrace ‘Krishnapuram’ bulb using HPLC-ESI-ITMS. J Biosci. 2021;46:1–7.

21. Xiao X, Ren W, Zhang N, Bing T, Liu X, Zhao Z, et al. Comparative Study of the Chemical Constituents and Bioactivities of the Extracts from Fruits, Leaves and Root Barks of Lycium barbarum. Molecules. 2019;24:1–22.

22. Campone L, Celano R, Piccinelli AL, Pagano I, Carabetta S, Sanzo R Di, et al. Response surface methodology to optimize supercritical carbon dioxide/co-solvent extraction of brown onion skin by-product as source of nutraceutical compounds. Food Chem. 2018;269 March:495–502.

23. Celano R, Docimo T, Piccinelli AL, Gazzerro P, Tucci M, Di Sanzo R, et al. Onion peel: Turning a food waste into a resource. Antioxidants. 2021;10:1–18.

24. Farag MA, Sakna ST, El-Fiky NM, Shabana MM, Wessjohann LA. Phytochemical, antioxidant and antidiabetic evaluation of eight Bauhinia L. species from Egypt using UHPLC-PDA-qTOF-MS and chemometrics. Phytochemistry. 2015;119:41–50.

25. Deladino L, Alvarez I, De Ancos B, Sánchez-Moreno C, Molina-García AD, Schneider Teixeira A. Betalains and phenolic compounds of leaves and stems of Alternanthera brasiliana and Alternanthera tenella. Food Res Int. 2017;97 April:240–9.

26. Lee JH, Lee SJ, Park S, Jeong SW, Kim CY, Jin JS, et al. Determination of flavonoid level variation in onion (Allium cepa L.) infected by Fusarium oxysporum using liquid chromatography-tandem mass spectrometry. Food Chem. 2012;133:1653–7.

27. Zeng X, Su W, Bai Y, Chen T, Yan Z, Wang J, et al. Urinary metabolite profiling of flavonoids in Chinese volunteers after consumption of orange juice by UFLC-Q-TOF-MS/MS. J Chromatogr B. 2017;1061–1062:79–88.

28. Cuyckens F, Claeys M. Mass spectrometry in the structural analysis of flavonoids. J Mass Spectrom. 2004;39:1–15.

29. Ghallab DS, Mohyeldin MM, Shawky E, Metwally AM, Ibrahim RS. Chemical profiling of Egyptian propolis and determination of its xanthine oxidase inhibitory properties using UPLC–MS/MS and chemometrics. Lwt. 2021;136:1–16.

30. Liu R, Ye M, Guo H, Bi K, Guo DA. Liquid chromatography/electrospray ionization mass spectrometry for the characterization of twenty-three flavonoids in the extract of Dalbergia odorifera. Rapid Commun Mass Spectrom. 2005;19:1557–65.

31. Abouzed TK, Contreras M del M, Sadek KM, Shukry M, H. Abdelhady D, Gouda WM, et al. Red onion scales ameliorated streptozotocin-induced diabetes and diabetic nephropathy in Wistar rats in relation to their metabolite fingerprint. Diabetes Res Clin Pract. 2018;140:253–64.

32. Sun K, Su C, Li W, Gong Z, Sha C, Liu R. Quality markers based on phytochemical analysis and anti-inflammatory screening: An integrated strategy for the quality control of Dalbergia odorifera by UHPLC-Q-Orbitrap HRMS. Phytomedicine. 2021;84 February:153511.

33. Yang K, Zhao Z, Gross RW, Han X. Identification and quantitation of unsaturated fatty acid isomers by electrospray ionization tandem mass spectrometry: A shotgun lipidomics approach. Anal Chem. 2011;83:4243–4250.

34. Li R, Zhou Y, Wu Z, Ding L. ESI-QqTOF-MS/MS and APCI-IT-MS/MS analysis of steroid saponins from the rhizomes of Dioscorea panthaica. J Mass Spectrom. 2006;41:1–22.
